# Dynamics of Membrane Potential Variation and Gene Expression Induced by *Spodoptera littoralis*, *Myzus persicae*, and *Pseudomonas syringae* in Arabidopsis

**DOI:** 10.1371/journal.pone.0046673

**Published:** 2012-10-30

**Authors:** Irene Bricchi, Cinzia M. Bertea, Andrea Occhipinti, Ivan A. Paponov, Massimo E. Maffei

**Affiliations:** 1 Plant Physiology Unit, Department of Life Sciences and Systems Biology, Innovation Centre, University of Turin, Turin, Italy; 2 Institut für Biologie II/Molecular Plant Physiology, Faculty of Biology, Albert-Ludwigs University of Freiburg, Freiburg, Germany; Instituto de Biología Molecular y Celular de Plantas, Spain

## Abstract

**Background:**

Biotic stress induced by various herbivores and pathogens invokes plant responses involving different defense mechanisms. However, we do not know whether different biotic stresses share a common response or which signaling pathways are involved in responses to different biotic stresses. We investigated the common and specific responses of *Arabidopsis thaliana* to three biotic stress agents: *Spodoptera littoralis*, *Myzus persicae*, and the pathogen *Pseudomonas syringae*.

**Methodology/Principal Findings:**

We used electrophysiology to determine the plasma membrane potential (V_m_) and we performed a gene microarray transcriptome analysis on Arabidopsis upon either herbivory or bacterial infection. V_m_ depolarization was induced by insect attack; however, the response was much more rapid to *S. littoralis* (30 min −2 h) than to *M. persicae* (4–6 h). *M. persicae* differentially regulated almost 10-fold more genes than by *S. littoralis* with an opposite regulation. *M. persicae* modulated genes involved in flavonoid, fatty acid, hormone, drug transport and chitin metabolism. *S. littoralis* regulated responses to heat, transcription and ion transport. The latest Vm depolarization (16 h) was found for *P. syringae*. The pathogen regulated responses to salicylate, jasmonate and to microorganisms. Despite this late response, the number of genes differentially regulated by *P. syringae* was closer to those regulated by *S. littoralis* than by *M. persicae*.

**Conclusions/Significance:**

Arabidopsis plasma membranes respond with a V_m_ depolarization at times depending on the nature of biotic attack which allow setting a time point for comparative genome-wide analysis. A clear relationship between V_m_ depolarization and gene expression was found. At V_m_ depolarization timing, *M. persicae* regulates a wider array of Arabidopsis genes with a clear and distinct regulation than *S. littoralis*. An almost completely opposite regulation was observed between the aphid and the pathogen, with the former suppressing and the latter activating Arabidopsis defense responses.

## Introduction

Plants are attacked by a multitude of organisms, like insects, microbes and fungi, which all infer a biotic stress. As plants are sessile organisms and cannot escape their predators they have evolved diverse mechanisms to react specifically to each attacking biotroph. Chewing herbivores, like *Spodoptera littoralis*, consume leaves by continuously clipping off and ingesting small pieces of tissue reducing both photosynthetic capacity and biomass of fed plants [1]–[5]. Aphids, like *Myzus persicae*, are sap-sucking insects that remove plant nutrients, elicit plant responses that are deleterious to plant productivity and alter the mass flow of phloem contents, resulting in changes in source–sink relationships [6]–[10]. Phytopathogenic bacteria of the genus *Pseudomonas* colonize the leaf surfaces of plants without causing disease [11]. *Pseudomonas syringae* multiplies in the plant cell apoplastic intercellular spaces and remains extracellular triggering plant defenses aimed to restrict bacterial growth [12].

Upon all of these biotic interactions with plants, it is crucial to understand how plants dissect and convert these different stress signals into appropriate physiological reactions.

The earliest event that is detectable as a consequence of leaf damage is depolarization of the plasma transmembrane potential (V_m_), followed by a cascade of biochemical and molecular events including protein phosphorylation, activation of signaling cascades and, eventually, gene expression and translation [12]–[17]. Both *S. littoralis* direct herbivory and the insect's oral secretions have been demonstrated to induce a fast mesophyll cell V_m_ depolarization of Arabidopsis [18] and other plant species [1], [19]–[21], whereas a significant V_m_ depolarization is observed at almost every stylet puncture of the plant plasmalemma during *M. persicae* phloem feeding [22]. In plant-pathogen interactions, V_m_ depolarization is a reliable early indicator of leaf hypersensitive response (HR) [23]

A variety of experimental methods have been employed to study the complex interactions of Arabidopsis and aphid herbivores, including measurements of the transcriptional responses [8], [24]–[27], whereas microarray-based genome-wide transcriptomic analyses have been performed in several plant species, including *Arabidopsis thaliana*, upon herbivore attack by *Spodoptera* spp., [28]–[31]. Although the exact nature of the systemic acquired resistance (SAR) signal in Arabidopsis after localized infection by avirulent *P. syringae* remains complex and has been a matter of debate [32], [33], the transcriptional changes associated with basal defense to live bacteria and the contribution of specific elicitors/effectors to the regulation of the basal defense transcriptome and other host physiological processes have been thoroughly studied [12], [34]–[36].

Although many commonly induced or suppressed defense-related genes have been identified in plants infested with chewing or phloem-feeding insects, and bacterial pathogens, there is considerable difference in the transcriptomic response of infested plants to different insects or bacteria. In the dazzling diversity of possible differential plant responses, the most difficult aspect is to assess whether a common response exists and to which extent each pathogen or herbivore differentially expresses and regulates defense response genes. Timing appears important in the interplay among the multiple plant responses to herbivores [37] and pathogenic microorganisms [38]. The aim of this work was to use a common physiological response to the herbivores *M. persicae* and *S. littoralis* and the pathogen *P. syringae* and, i.e. the leaf V_m_ depolarization, as a time point for a comparative genome-wide analysis of gene expression and regulation in Arabidopsis, when attacked by different biotic agents. The obtained results should complement other studies and provide a useful resource for future study of plant multitrophic interactions.

## Results

### *S. littoralis*, *M. persicae* and *P. syringae* induce the same strong V_m_ depolarization in *A. thaliana* leaves but at different times

**Figure 1:**
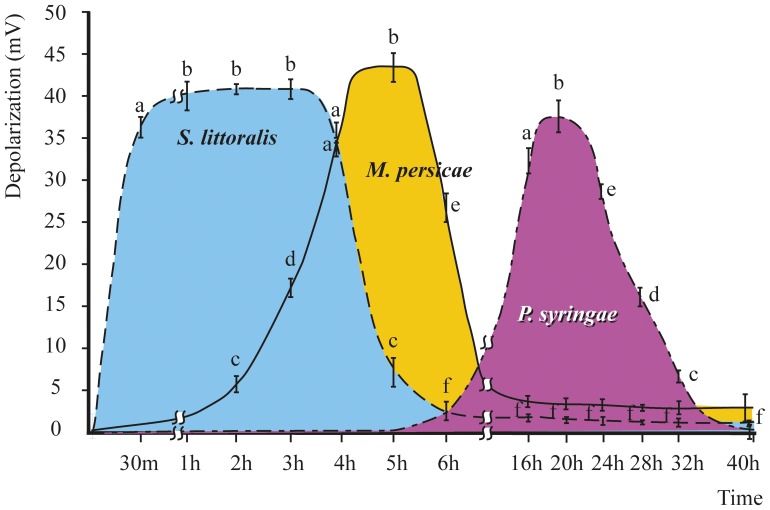
Plasma transmembrane potential (V_m_) depolarization measured in Arabidopsis mesophyll leaves at different times upon herbivory by *Spodoptera littoralis* and *Myzus persicae* and infection by *Pseudomonas syringae*. Chewing herbivore induces a fast V_m_ depolarization that lasts about 4–6 h from feeding, whereas phloem feeding induces a V_m_ depolarization that occurs after about 6 h from feeding. Infection by *P. syringae* causes a V_m_ depolarization about 16 h after infection. No matter the biotic stress the level of the highest V_m_ depolarization shows the same value (statistical significance P>0.05). For each time point at least 50 measurements were performed. The timing of V_m_ depolarization depends on biotic damage. Bars represent standard error, different letters indicate significant (P<0.05) differences.

Time-course measurements of V_m_ in Arabidopsis showed that after *S. littoralis* herbivory a strong and rapid V_m_ depolarization (with respect to mechanical damage) occurs after a few minutes from the herbivore wound, with recovery of the V_m_ between 5 and 6 h (Figure 1). When Arabidopsis was fed by *M. persicae*, almost the same extent of V_m_ depolarization was observed (P>0.05), but the timing of V_m_ depolarization peaked between 4 and 6 h, returning near to the basal V_m_ value between 16 and 24 h (Figure 1). A remarkable delay in V_m_ depolarization was observed when Arabidopsis leaves were infected by the avirulent strain of *P. syringae*. Even in this case V_m_ depolarization was not significantly different (P>0.05) from the values observed after *S. littoralis* and *M. persicae* herbivory; however, the maximal V_m_ depolarization occurred between 16 and 18 h from inoculation (Figure 1). These results indicate that Arabidopsis responds to different biotic stress with a strong and transient V_m_ depolarization and that the timing of this event depends on the kind of biotic stress.

### Comparative gene expression at the time of V_m_ depolarization in Arabidopsis leaves infested by *S. littoralis*, *M. persicae* and *P. syringae*

Taking into account that different biotrophs, owing to their peculiar feeding or infesting behavior, can cause different amounts of damage or trigger plant responses at different time, samples for microarray analysis were taken at time points corresponding to the maximum V_m_ depolarization for every biotic stress.

To identify transcriptional changes in Arabidopsis, total RNA was extracted from leaves wounded for 2 h by the herbivore *S. littoralis*, for 5 h by the phloem feeder *M. persicae* and after 16 h from inoculation of the pathogen *P. syringae*, these timings corresponding to maximal V_m_ depolarization. As control for *S. littoralis*, leaves were mechanically damaged with a pattern wheel, whereas for *M. persicae* leaves were wounded with the tip of an electrophysiology microcapillary. Mechanical damage was done at the same extension as observed after herbivory. Control for *P. syringae* consisted of MgCl_2_ leaf infiltration.

**Figure 2:**
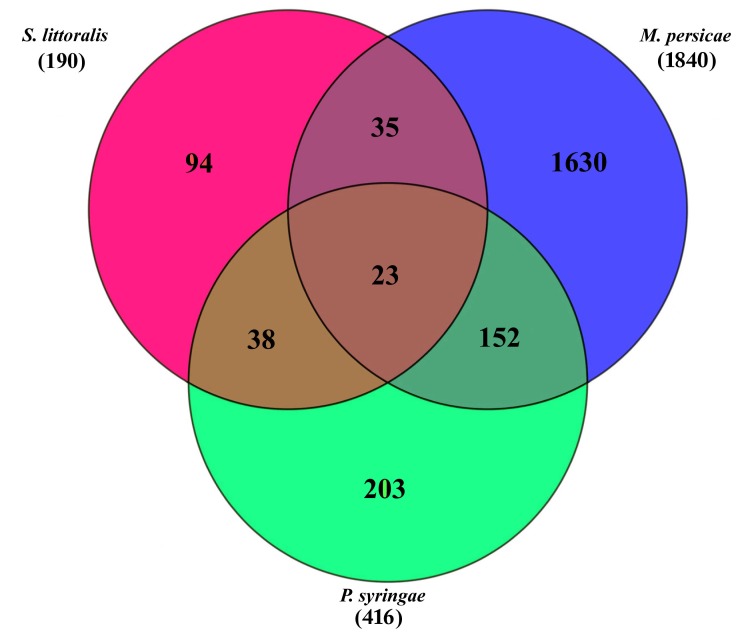
Venn diagram of commonly and differentially expressed Arabidopsis genes upon herbivory by *Spodoptera littoralis* and *Myzus persicae* and infection by *Pseudomonas syringae*.

In order to evaluate robustly regulated sequences that are useful to evaluate the plant response to biotic stress, four biological replicates of each biotic stress (each consisting of several stressed leaves) were used for the gene microarray analysis. By using the stringent criteria described in material and methods (fold change ≥2, P≤0.05), out of 38,463 sequences on the Agilent spotted slide, 190 genes fulfilled these stringent criteria for *S. littoralis*, 1840 genes for *M. persicae* and 416 for *P. syringae*. Among these genes, 23 were commonly regulated, whereas the comparative analysis between the biotrophs revealed the presence of 35, 38 and 152 co-regulated genes in the interactions *M. persicae/S. littoralis*, *S. littoralis/P. syringae* and *M. persicae/P. syringae*, respectively (Figure 2).

### A few Arabidopsis commonly expressed genes are regulated upon *M. persicae* and *S. littoralis* herbivory and *P. syringae* infection

**Figure 3:**
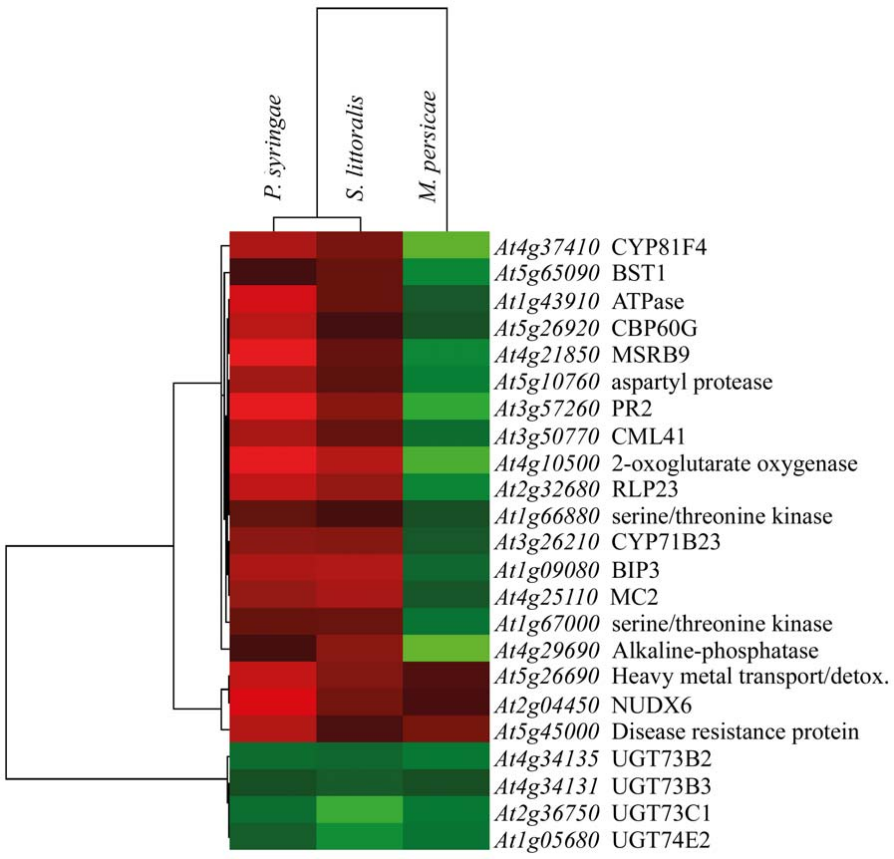
Cluster analysis of commonly regulated genes in Arabidopsis fed by the herbivores *Myzus persicae* and *Spodoptera littoralis* and infected by *Pseudomonas syringae*. *M. persicae* shows a lower statistical linkage with *S. littoralis* and *P. syringae*, which are linked together.

The cluster analysis of commonly regulated genes shows that at the time of V_m_ depolarization a common feature of Arabidopsis plants under biotic stress is a cluster of down-regulated genes including four UDP-glycosyltransferases (*UGT73B2*, *UGT73B3*, *UGT73C1*, *UGT74E2*) and a cluster of up-regulated genes made of Toll-Interleukin-Resistance (TIR-NBS-LRR class) transmembrane receptor (*At5g45000*), NADH pyrophosphatase (*NUDX6*) and heavy metal transport/detoxification superfamily protein (*At5g26690*) (Figure 3 and Table 1). All other commonly regulated genes were differentially expressed and the cluster analysis showed a close linkage between *P. syringae* and *S. littoralis*, indicating a common up-regulation of the remaining genes, whereas the same genes were always down-regulated by *M. persicae* (Table 1 and Figure 3).

**Table 1:** *Arabidopsis thaliana* genes commonly expressed at the time of Vm depolarization upon *Spodoptera littoralis* (2 h) and *Myzus persicae* (5 h) herbivory and *Pseudomonas syringae* (16 h) infection.

GO category and AGI	Short description	*P. syringae*	*S. littoralis*	*M. persicae*
**Transferase and Transporter activity**				
At4g34135	flavonol 7-O-glucosyltransferase (Group D) (UGT73B2)	−3.00	−2.82	−3.40
At4g34131	UDP-glucosyl transferase (Group D) (UGT73B3)	−2.22	−2.53	−2.23
At2g36750	UDP-glucosyl transferase (Group D) (UGT73C1)	−3.08	−6.66	−3.40
At1g05680	UDP-glucosyltransferase (Group L) (UGT74E2)	−2.59	−4.21	−3.23
**Hydrolase activity**				
At1g43910	P-loop containing nucleoside triphosphate hydrolases superfamily protein	8.83	2.90	−2.43
At3g57260	beta 1,3-glucanase (PR2)	14.85	3.94	−6.05
At4g29690	Alkaline-phosphatase-like family protein	2.14	4.06	−14.95
At5g65090	involved in root hair morphogenesis and tip growth (BST1)	2.09	2.91	−3.89
**Kinase activity**				
At1g66880	protein serine/threonine kinase activity	2.74	2.14	−2.23
At1g67000	protein serine/threonine kinase activity	2.87	2.97	−3.24
At2g32680	receptor like protein 23 (RLP23)	7.11	4.55	−3.85
**Response to biotic stress**				
At2g04450	NADH pyrophosphatase (NUDX6)	9.33	3.28	2.20
At5g26690	Heavy metal transport/detoxification superfamily protein	7.42	3.74	2.34
At5g45000	Disease resistance protein (TIR-NBS-LRR class) family	6.20	2.26	3.39
At1g09080	ATP binding protein (BIP3)	5.76	6.05	−2.84
At3g26210	cytochrome P450 (CYP71B23)	4.15	3.86	−2.43
At4g37410	cytochrome P450 (CYP81F4)	5.82	3.42	−11.95
At3g50770	calmodulin-like 41 (CML41)	5.71	2.84	−3.01
At5g26920	calmodulin-binding protein (CBP60G)	6.61	3.74	−2.30
At4g10500	2-oxoglutarate (2OG) and Fe(II)-dependent oxygenase superfamily protein	33.52	6.41	−8.40
At4g21850	methionine sulfoxide reductase (MSRB9)	23.57	2.81	−3.91
At4g25110	type I metacaspase (MC2)	4.55	5.59	−2.42
At5g10760	aspartyl protease family protein	5.09	2.61	−3.58

Values are expressed as fold change with respect to controls (P<0.05). AGI, Arabidopsis Genome Initiative gene index.

### Commonly expressed Arabidopsis genes upon *S. littoralis* and *M. persicae* herbivory show different regulation patterns

**Figure 4:**
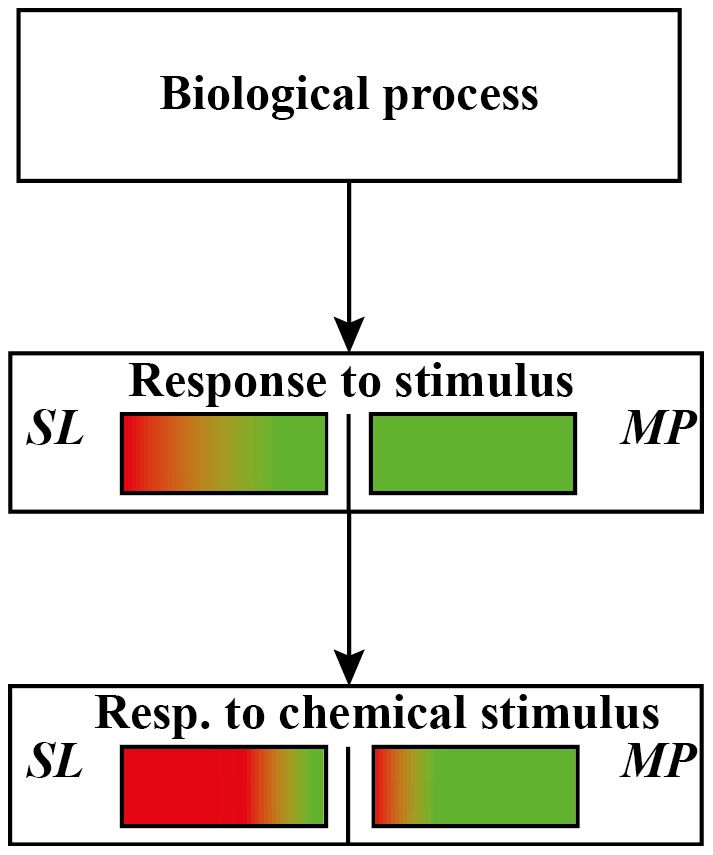
GO analysis of Arabidopsis commonly expressed genes upon *Spodoptera littoralis* (*SL*) and *Myzus persicae* (*MP*) herbivory. Red color indicates up-regulation, green color indicates down-regulation.

Most of the genes that were commonly regulated by *M. persicae* and *S. littoralis* herbivory showed opposite regulation, with many genes showing down-regulation upon *M. persicae* feeding and up-regulation after *S. littoralis* herbivory (Figure 4 and Table 2).

**Table 2:** *Arabidopsis thaliana* genes commonly expressed at the time of Vm depolarization upon *Spodoptera littoralis* (2 h) and *Myzus persicae* (5 h) herbivory.

GO categories	AGI	Short description	*M. persicae*	*S. littoralis*
**Response to stimulus**				
Response to stimulus	At1g76690	12-oxophytodienoic acid reductase (OPR2)	−2.57	−2.43
	At5g41750	disease resistance protein (TIR-NBS-LRR class)	−2.63	2.63
Response to chemical stimulus	At1g66370	MYB113	−5.81	4.30
	At3g59220	cupin-domain containing protein (PRN1)	−6.31	−2.98
	At3g23240	ethylene response factor (ERF1)	−4.95	2.44
	At5g47220	ethylene response factor (ERF2)	−3.45	3.07
	At1g77120	alcohol dehydrogenase (ADH1)	2.41	−2.26
	At5g17330	glutamate decarboxylase (GAD1)	−2.40	4.12
	At5g24380	metal-phytosiderophore yellow stripe like (YSL2)	−2.11	2.94
**Other GO categories**				
Transferase and Transporter activity	At2g37870	protease inhibitor/seed storage/lipid transfer protein (LTP) family protein	−2.83	−3.35
	At3g50280	HXXXD-type acyl-transferase family protein	−3.23	2.01
	At5g54060	UDP-glucose:flavonoid 3-o-glucosyltransferase (UF3GT)	−12.36	3.99
Hydrolase activity	At1g14890	invertase/pectin methylesterase inhibitor superfamily protein;	2.41	−2.02
	At4g29700	Alkaline-phosphatase-like family protein;	−2.38	2.62
	At1g53830	pectin methylesterase (PME2)	−4.09	2.36
	At3g62040	Haloacid dehalogenase-like hydrolase (HAD) superfamily protein	−5.13	2.35
Transcription factors	At3g48360	essential component of the TAC1-mediated telomerase activation pathway (BT2)	8.63	2.78
	At5g22380	ANAC090	−16.79	4.78
Response to biotic stress	At4g36850	PQ-loop repeat family protein/transmembrane family protein	25.48	5.22
	At3g13520	GPI-anchored arabinogalactan peptide (AGP12)	3.36	−2.54
	At1g77120	alcohol dehydrogenase (ADH1)	2.41	−2.26
	At3g30460	zinc finger (C3HC4-type RING finger) family protein	2.33	−2.35
	At4g01575	serine protease inhibitor, Kazal-type family protein	2.26	−2.19
	At5g02540	short-chain dehydrogenase/reductase (SDR) family protein	2.15	−2.14
	At4g37540	LOB domain-containing protein (LBD39)	2.01	2.07
	At5g07460	Methionine sulfoxide reductase (ATMSRA2)	−2.08	2.01
	At4g24350	phosphorylase family protein	−2.09	2.12
	At2g25130	armadillo/beta-catenin repeat family protein	−2.46	−3.17
	At5g53820	Late embryogenesis abundant protein (LEA) family protein	−2.59	−2.04
	At1g59590	ZCF37 mRNA	−2.66	4.10
	At2g27310	F-box family protein	−3.18	2.06
	At2g47560	zinc finger (C3HC4-type RING finger) family protein	−3.31	3.14
	At1g24140	matrixin family protein metallopeptidase activity, metalloendopeptidase activity	−3.88	2.52
	At1g13470	unknown protein	−2.11	6.16
	At5g22545	unknown protein	−2.37	2.84

Values are expressed as fold change with respect to controls (P<0.05). AGI, Arabidopsis Genome Initiative gene index.

Genes annotated for response to stimulus showed the same expression trends between the two herbivores for *OPR2* and *PRN1*, which were commonly down-regulated, whereas up-regulation by *S. littoralis* and down-regulation by *M. persicae* was found for ethylene response factors (*ERF1*, *ERF2*), *MYB113*, *GAD1* and a metal-phytosiderophore (*YSL2*). *S. littoralis* down-regulated alcohol dehydrogenase (*ADH1*), which was up-regulated by *M. persicae*.

An opposite gene regulation, with up-regulation caused by *S. littoralis*, was also found for several genes belonging to other GO categories like transferases (*UF3GT*, *At3g50280*), hydrolases (*PME2*, *HAD*, *At4g29700*), transcription factors (TFs) (*ANAC090*) and some genes responding to biotic stress (Table 2). On the other hand, an opposite regulation, with up-regulation caused by *M. persicae*, was found for an invertase/pectin methylesterase inhibitor (*At2g37870*), a GPI-anchored arabinogalactan peptide (*AGP12*), ADH1, and some other genes responding to biotic stress (Table 2). A common up-regulation was found for an essential component of the TAC1-mediated telomerase activation pathway (*BT2*), a PQ-loop repeat family protein/transmembrane family protein (*At4g36850*) and methionine sulfoxide reductase (*ATMSRA2*). Both insects down-regulated protease inhibitor/seed storage/lipid transfer protein (*At2g37870*), late embryogenesis abundant protein (*LEA*) (*At5g53820*) and armadillo/beta-catenin repeat family protein (*At2g25130*).

### Commonly expressed Arabidopsis genes upon *S. littoralis* herbivory and *P. syringae* infection show the same regulation pattern

**Figure 5:**
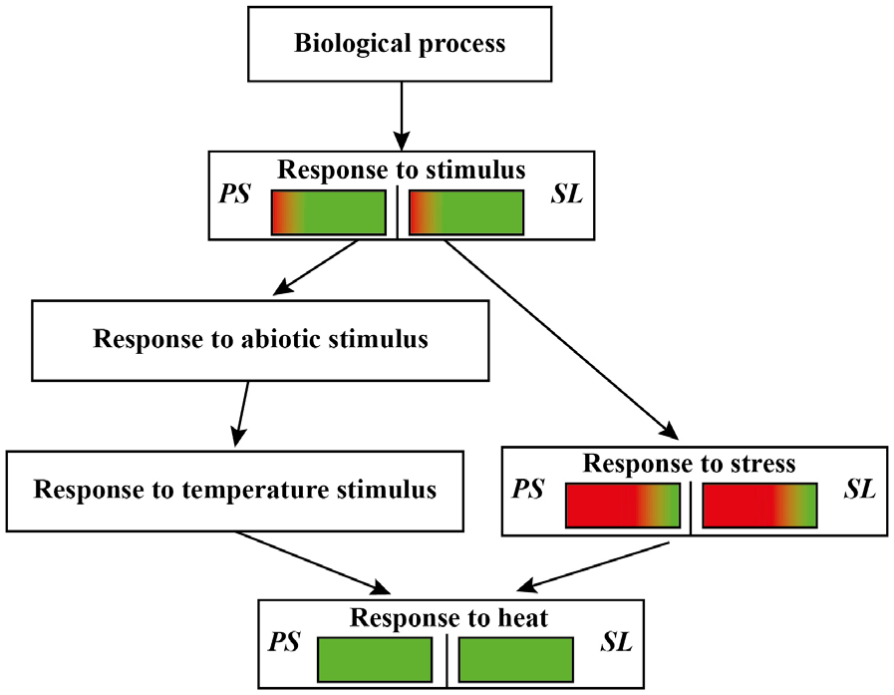
GO analysis of Arabidopsis commonly expressed genes upon *Pseudomonas syringae* (*PS*) infection and *Spodoptera littoralis* (*SL*) herbivory. Red color indicates up-regulation, green color indicates down-regulation.

The GO analysis of genes commonly expressed in Arabidopsis upon infection of the pathogen *P. syringae* and the chewing herbivore *S. littoralis* classified 35 of the 38 annotated genes. Most of them were related to response to stimulus, leading to a common response to heat (Figure 5).

Several small heat shock proteins (sHSPs) (*HSP17*, *HSP17.6II*, *HSP17.6A-CI*, *HSP17.6*, *HSP21*, *HSP23.6-MITO*) and *HSP70* were down-regulated by both *P. syringae* and *S. littoralis* biotic stress, as were genes involved in glutathione *S*-transferases (*GST20*, *GSTU24*), UDP-glycosyltransferase (*UGT73B4*) and a TF (*ANAC102*). Most of the remaining commonly expressed genes were up-regulated by both *S. littoralis* and *P. syringae* (Table 3). A remarkable up-regulation was found upon *P. syringae* infection for a beta-1,3-glucanase (*BG3*), triacylglycerol lipase (*At5g24200*) and genes related to ion binding and resistance (*At3g57460*, *PCR1*).

**Table 3:** *Arabidopsis thaliana* genes commonly expressed at the time of Vm depolarization upon *Spodoptera littoralis* (2 h) herbivory and *Pseudomonas syringae* (16 h) infection.

GO categories	AGI	Short description	*P. syringae*	*S. littoralis*
**Response to stimulus**				
Response to temperature (heat)	At5g12030	Small Heat Shock Protein (HSP17)	−12.75	−22.62
	At5g12020	Small Heat Shock Protein (HSP17.6II)	−16.00	−10.05
	At1g07400	Small Heat Shock Protein (HSP17.6A-CI)	−5.27	−2.60
	At1g53540	Small Heat Shock Protein (HSP17.6)	−6.06	−18.65
	At1g52560	Small Heat Shock Protein (HSP21)	−10.12	−36.21
	At4g25200	Small Heat Shock Protein (HSP23.6-MITO)	−15.13	−23.74
	At5g51440	Small Heat Shock Protein (HSP23.6-MITO)	−5.46	−3.89
	At1g16030	Heat Shock Protein (HSP70)	−4.62	−6.24
Response to stress	At1g76680	12-oxophytodienoic acid reductase (OPR1)	−2.04	−2.18
	At3g01080	WRKY58	3.47	4.55
	At3g45860	cysteine-rich receptor-like protein kinase (CRK4)	3.45	3.47
	At4g04220	Receptor Like Protein (RLP46)	2.17	2.04
Other responses to stimulus	At3g57240	beta-1,3-glucanase (BG3)	26.39	3.20
	At1g77760	nitrate reductase (NR1)	2.24	5.20
	At5g24530	downy mildew resistant (DMR6) oxygenase	4.09	2.71
	At3g50480	Homolog of RPW8 (HR4)	3.71	2.17
	At2g29480	glutathione S-transferase (GST20)	−4.06	−4.38
	At1g17170	glutathione S-transferase (GSTU24)	−6.05	−5.08
	At2g15490	UDP-glycosyltransferase (UGT73B4)	−16.45	−6.81
**Other GO categories**				
Hydrolase activity	At5g24200	triacylglycerol lipase	20.36	3.14
Kinase activity	At1g35710	leucine-rich repeat transmembrane protein kinase, putative	4.34	3.52
	At5g59680	leucine-rich repeat protein kinase, putative	2.93	6.16
Transcription factors	At5g63790	NAC family of transcription factors (ANAC102)	−2.05	−2.16
	At5g01900	WRKY62	9.52	3.31
	At3g11580	AP2/B3-like transcription factor	6.03	−2.08
Other response to biotic stress	At3g57460	catalytic/metal ion binding/metalloendopeptidase/zinc ion binding	28.85	3.68
	At1g14880	plant cadmium resistance (PCR1)	21.36	7.39
	At5g39670	calcium-binding EF hand family protein	11.87	2.45
	At3g47480	calcium-binding EF hand family protein	7.87	2.65
	At5g55460	protease inhibitor/seed storage/lipid transfer protein (LTP) family protein	6.97	4.87
	At1g10340	ankyrin repeat family protein	6.72	2.87
	At4g03450	ankyrin repeat family protein	5.44	3.23
	At2g47130	short-chain dehydrogenase/reductase (SDR3)	5.00	2.96
	At1g23840	unknown protein located in endomembrane system	3.50	2.97
	At3g61280	unknown protein	3.45	2.28
	At3g48640	unknown protein	5.77	3.00
	At3g61920	unknown protein	2.41	−2.04

Values are expressed as fold change with respect to controls (P<0.05). AGI, Arabidopsis Genome Initiative gene index.

### Commonly expressed Arabidopsis genes upon *M. persicae* herbivory and *P. syringae* infection reveal a remarkable opposite regulation

**Figure 6:**
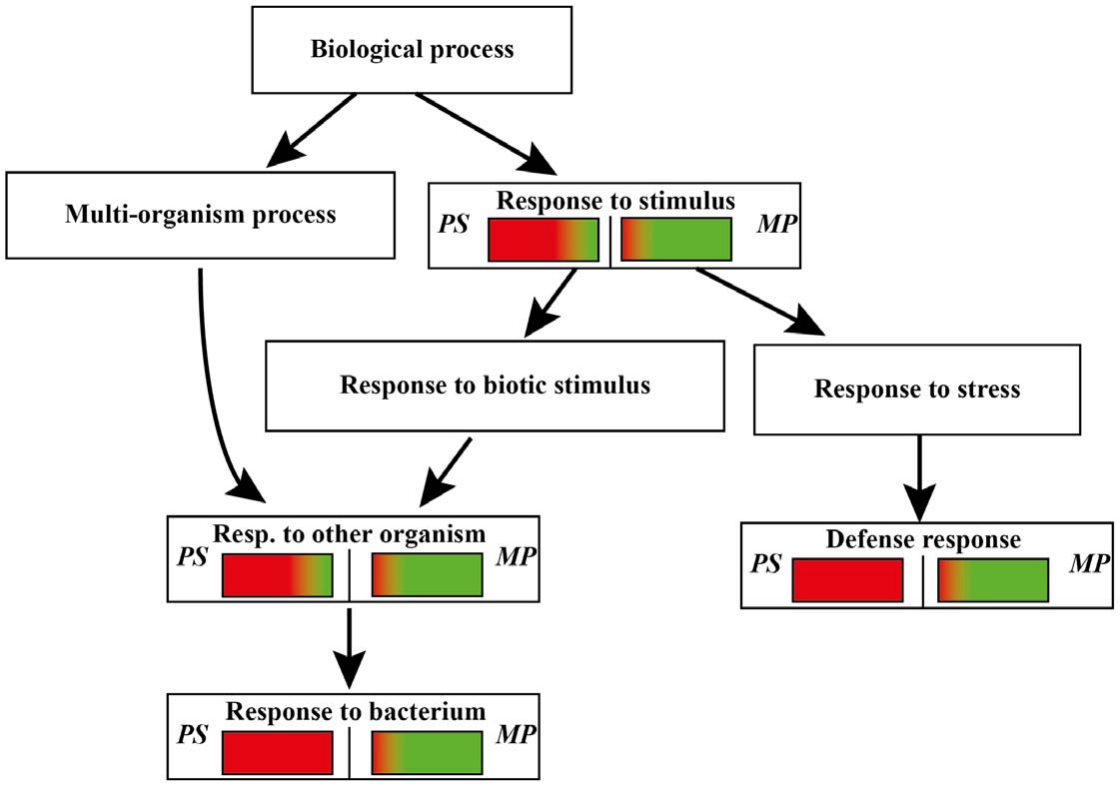
GO analysis of Arabidopsis commonly expressed genes upon *Pseudomonas syringae* (*PS*) infection and *Myzus persicae* (*MP*) herbivory. Red color indicates up-regulation, green color indicates down-regulation.

The GO analysis of genes commonly expressed in Arabidopsis upon infection of the pathogen *P. syringae* and the phloem feeding *M. persicae* classified 135 genes among 152 annotated genes. Among these annotated genes, several were typical of responses to bacterium, response to other organisms, and response to stimulus (Figure 6 and Table S1)

Up-regulation after both *M. persicae* and *P. syringae* biotic stress was found for genes related to pathogenesis (*NIMIN-1*, *PCC1*, *ACD6*, *PR5*, *PR13*), a receptor like protein (*RLP38*), an S-locus lectin protein kinase family protein (*At1g11330*), glutaredoxin (*GRXS13*), a gene up-regulated in response to *Hyaloperonospora parasitica* (*LURP1*), genes involved in SA and JA metabolism (*ANK, BSMT1, MES9, JMT, EDS5*), a NPR1/NIM1-interacting gene (*NIMIN-2*) and a TF (*WRKY38*).

A common down-regulation was found for a glucosyl transferase (*UGT73B5*), an ABC transporter (*ABCB4*) and a nodulin MtN21 family protein (*At1g70260*).

An opposite regulation was found for several receptor-like proteins (*RLP41*, *RLP43*), cysteine-rich receptor-like protein kinases (*CRK6, CRK7, CRK23, CRK36*), flg22-induced receptor-like kinase 1 (*FRK1*), a putative receptor serine/threonine kinase (*At4g18250*), a lectin receptor kinase (*At5g01550*), genes responding to hormone stimulus (*JAZ10*, *GID1B* and *At3g12830*), several transporters (*GPT2, LHT7, At3g21080, ST2A, PTR3*), genes coding for hydrolase activity (*CWINV6, CHI, At1g54010*), a kinase (*MAPKKK19*), a TF (*WRKY55*) and several other genes expressed in response to biotic stress (*At1g14120*, *At3g49340*, *MSRB8*, *LAS1*) (Table S1). Several other genes showing opposite trends are listed in Table S1.

### Differential gene expression in Arabidopsis upon *S. littoralis* herbivory reveals up-regulation of genes involved in transcription regulation and down-regulation of heat-shock proteins

**Figure 7:**
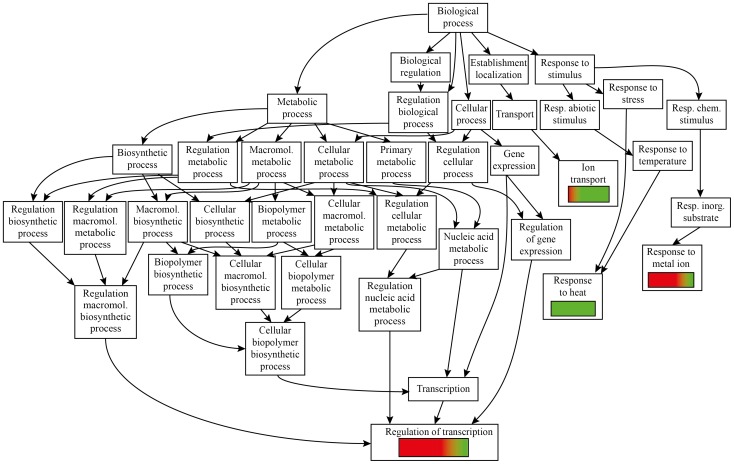
GO analysis of Arabidopsis genes regulated by *Spodoptera littoralis* herbivory. Red color indicates up-regulation, green color indicates down-regulation.

Among the 94 genes specifically regulated by *S. littoralis* herbivory, 78 were annotated and GO analysis isolated four gene categories: genes involved in the regulation of transcription, response to heat, ion transport and response to metal ion (Figure 7)

In the regulation of transcription category, most of the genes were up-regulated by *S. littoralis* herbivory, including several genes involved in the circadian clock, whereas two basic helix-loop-helix (bHLH) (*At4g20970*, *ATBHLH096*) and a MYB like DNA-binding protein (*AtGT-3a*) were down-regulated (Table 4).

**Table 4:** *Arabidopsis thaliana* genes differentially expressed at the time of Vm depolarization (2 h) upon *Spodoptera littoralis* herbivory.

GO Category	AGI	Short description	FC (P<0.05)
Regulation of transcription	*At4g06746*	DREB subfamily A-5 of ERF/AP2 transcription factor family (RAP2.9)	−3.17
	*At2g22200*	DREB subfamily A-6 of ERF/AP2 transcription factor family.	2.49
	*At4g20970*	basic helix-loop-helix (bHLH) family protein	−3.40
	*At1g72210*	ATBHLH096	−2.48
	*At4g05170*	basic helix-loop-helix (bHLH) family protein	3.19
	*At2g28160*	ATBHLH029	2.56
	*At3g09600*	MYB TF (LHY-CCA1-LIKE5)	2.41
	*At2g21650*	MYB TF (AtRL2 - RAD-LIKE 2)	2.09
	*At5g17300*	MYB TF (REVEILLE 1)	2.94
	*At1g01060*	MYB TF (LATE ELONGATED HYPOCOTYL 1, LHY1)	3.80
	*At2g46830*	MYB TF (CIRCADIAN CLOCK ASSOCIATED 1, CCA1)	2.05
	*At5g01380*	MYB like DNA-binding protein (AtGT-3a)	−2.18
	*At1g69570*	Dof-type zinc finger domain-containing protein	2.28
	*At3g21150*	zinc finger TF (B-box type ATBBX32)	2.95
	*At1g26960*	homeodomain leucine zipper class I (HD-Zip I, ATHB23)	2.33
response to heat	*At3g46230*	17.4 kDa class I heat shock protein (HSP17.4-CI)	−20.03
	*At1g59860*	17.8 kDa class I heat shock protein (HSP17.8-CI)	−8.90
	*At2g29500*	17.8 kDa class I heat shock protein (HSP17.8-CI)	−4.71
	*At1g74310*	ClpB1, Also known as AtHsp101.	−3.25
	*At4g21320*	heat-stress-associated 32-kD protein	−2.20
	*At2g32120*	HSP70 family protein (Hsc70.1)	−3.33
	*At3g61190*	protein with a C2 domain that binds to BON1 (BAP1)	−2.67
	*At4g12400*	stress-inducible protein	−2.26
Response to metal ion	*At3g56240*	copper chaperone CCH protein	2.02
	*At2g40300*	Ferritin-4 (FER4)	−2.49
Ion transport	*At3g63380*	calcium-transporting ATPase 12, plasma membrane-type (ACA12)	−2.95
	*At3g22910*	calcium-transporting ATPase 13, plasma membrane-type (ACA13)	−2.20
	*At3g56240*	CCH protein	2.01
	*At2g04032*	Zinc transporter (ZIP7)	−2.24
	*At1g77990*	sulfate transporter	2.40

Values are expressed as fold change with respect to controls (P<0.05). AGI, Arabidopsis Genome Initiative gene index.

In the response to heat category, all expressed sHSPs and HSPs were down-regulated, whereas in response to metal ion, a copper chaperone CCH protein (*At3g56240*) was up-regulated and a ferritin (*FER4*) was down-regulated (Table 4). In the ion transport category, two Ca^2+−^ATPase (*ACA12, ACA13*) and a zinc transporter (*ZIP7*) were down-regulated, whereas a copper (*At3g56240*) and a sulfate (*At1g77990*) transporters were up-regulated.

Table S2 reports values of all other differentially expressed genes upon *S. littoralis* herbivory.

### Differential gene expression in Arabidopsis leaves upon *M. persicae* herbivory reveals down-regulation of secondary metabolism and up-regulation of cell wall components

**Figure 8:**
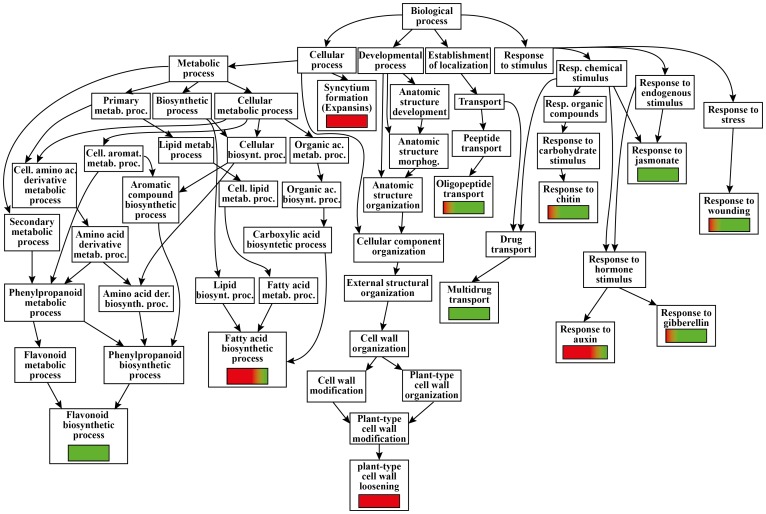
GO analysis of Arabidopsis genes regulated by *Myzus persicae* herbivory. Red color indicates up-regulation, green color indicates down-regulation.

Among the 1630 Arabidopsis differentially expressed genes upon *M. persicae* herbivory, 1423 were annotated and GO analysis classified 10 gene categories (Figure 8).

A strong down-regulation was found for many genes related to flavonoid biosynthetic process (*DFR*), particularly those related to metabolism of anthocyanin/anthocyanidin (*ANS*, *5MAT*, *At1g03940*, *At4g22870*, *MYB75*, *MYB123*), and flavonoid hydroxylation (*CYP75B1*, *F3H*) and isomerization (*CFI*, *At5g05270*). The only up-regulation were found for a chalcone-flavanone isomerase family protein (*At1g53520*) and a gene involved in brassinosteroid metabolic pathway (*BEN1*) (Table S3). Genes involved in fatty acid metabolism were all up-regulated, with the only exception of 4-coumarate-CoA ligase-like (*4CLL5*), that was down-regulated.

Most of genes involved in fatty acid biosynthetic process were up-regulated (particularly a Δ-9 desaturase-like 5 protein, *At1g06360*), whereas a strong down-regulation was found for a P450-dependent fatty acid omega-hydroxylase (*At3g48520*).

Genes involved in cell wall modification and loosening included 11 expansin genes, 10 of which were up-regulated by *M. persicae* feeding (particularly *EXP8*), whereas an expansin-related gene (*EXLB1*) was down-regulated (Table S3).

Aphid feeding regulated oligopeptide transport with many genes being either up-regulated or down-regulated. On the other hand, almost all multidrug transport-related genes belonging to the MATE efflux family were down-regulated. Aphid feeding repressed Arabidopsis TF responding to chitin, including several WRKY (*WRKY18*, *WRKY40*, *WRKY48*, *WRKY53*, *WRKY60*), MYB (*MYB31*, *MYB44*, *MYB59*), members of the NAC TF family (*ANAC036*, *ANAC061*), zinc finger proteins (*CZF1*, *CZF1*/*ZFAR1*, *AZF2*, *AZF3*, *STZ*), ethylene response factors (*ERF1*, *RRTF1*, *ERF13*), members of the DREB subfamily A-6 of ERF/AP2 TFs (*DEAR19*, *At4g28140*), scarecrow-like (*SCL13*), salt tolerance homolog (*STH2*), armadillo-like (*At2g35930*) and RING-H2 finger protein (*ATL5H*). A response regulator (*ARR7*), a U-box domain-containing protein similar to immediate-early fungal elicitor (*At3g18710*) and *WRKY17* were up-regulated (Table S3).

Genes responding to wounding were either up-regulated (*RLM3*, *At5g57170*, *5PTASE13*) or down-regulated, like a terpene synthase involved in (*E*)-β-ocimene synthesis (*TPS03*).

*M. persicae* also strongly regulated genes involved in action of three important hormone classes: auxins, gibberellins and JA.

Four auxin induced proteins (*IAA5*, *IAA6*, *IA14*, *IAA5*), an auxin influx transporter (*AUX1*), an auxin resistant gene (*AXR2*), 8 small auxin up-regulated (SAUR) responsive proteins (*SAUR15*, *SAUR19*, *SAUR20*, *SAUR22*, *SAUR23*, *SAUR26*, *SAUR29*, *SAUR68*), and 12 SAUR-like auxin-responsive proteins were up-regulated (Table S3). Up-regulation was also found for a IAA-amido synthetase (*GH3.9*) and a nucleotide diphosphate kinase (*NDPK2*), whereas MYB95 and a MYB responding to auxin stimulus were down-regulated.

GA-regulated proteins (particularly *GASA6*) and GA oxidases (*GA3OX1*, *GA20OX1*) were all up-regulated, whereas down-regulation was found for a GA-regulated protein (*GASA5*), a DELLA protein (*RGL3*), *MYB7* and α-amylase (*AMY1*).

All genes responding to JA were down-regulated by *M. persicae* feeding, including genes involved in JA metabolism (*OPR3*, *AOC3*, *CYP94B3*), 8 JA-Zim-domain proteins (*JAZ1*, *JAZ2*, *JAZ3*, *JAZ5*, *JAZ6*, *JAZ7*, *JAZ9*, *JAZ10*), 2 JA responsive proteins (*JR1*, *JR2*), lipoxygenases (*LOX3*, *LOX4*), *MYC2*, *MYB47*, *MYB74*, syntaxin (*SYP122*) and, particularly, ribonuclease T2 (*RNS1*) and a thionin (*THI2.1*) (Table S3).

The full list of all other genes regulated by *M. persicae* feeding can be found in Table S4.

### Differential gene expression in Arabidopsis leaves upon *P. syringae* infection shows up-regulation of defense genes including responses to JA and SA

**Figure 9:**
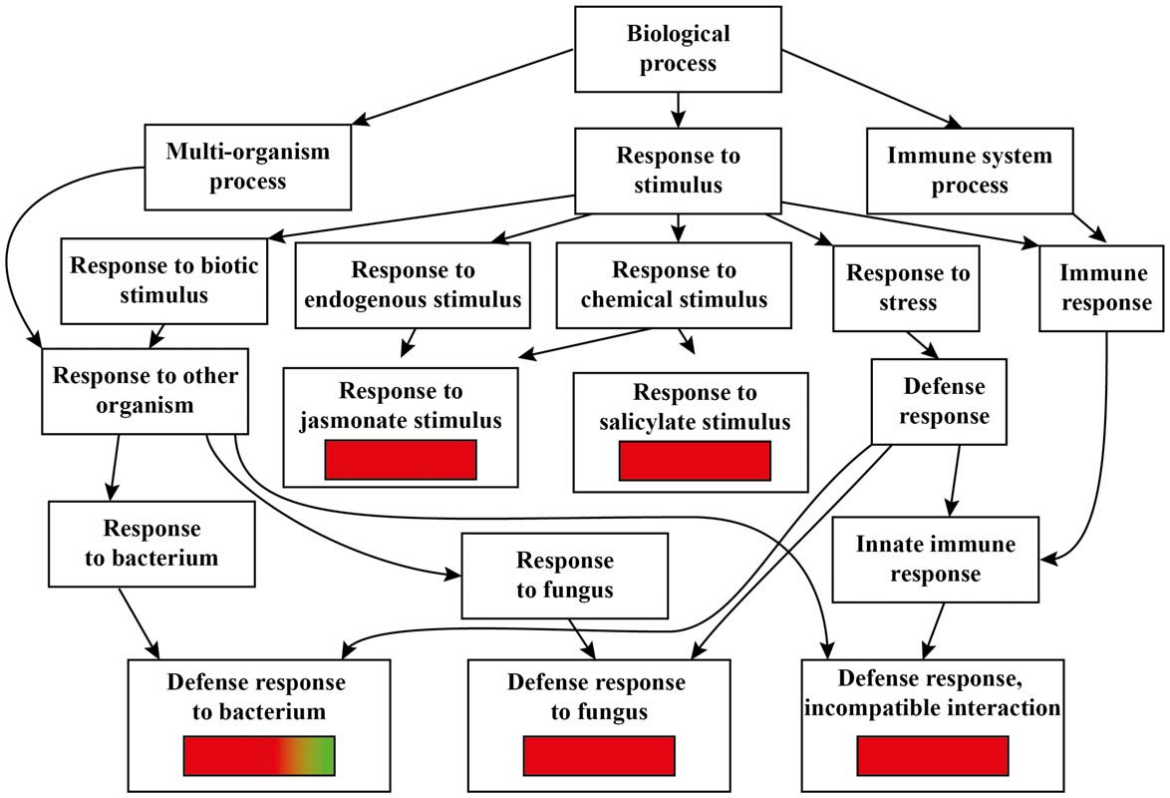
GO analysis of Arabidopsis genes regulated by *Pseudomonas syringae* infection. Red color indicates up-regulation, green color indicates down-regulation.

Among the 203 genes specifically regulated by *P. syringae* herbivory, 186 were annotated and GO analysis identified three gene categories: defense genes in response to bacterium, to fungus and incompatible interaction, and response genes to JA and SA (Figure 9)

Almost all differentially expressed genes in the defense response to bacterium were up-regulated with the only exception of a plasma membrane polypeptide (*ATPCAP1*) (Table 5). Up-regulation was also found for all genes responsive to fungus (*At2g14560*, *At4g12490*, *At2g39200*) and in incompatible interaction (*PDF1.2A, AOC2*), the latter two genes being also involved in responses to SA and JA (Table 5). Genes responsive to SA included two kinases (*CRK9, WAK1*) and a phytoalexin-deficient 4 protein (*PAD4*). In response to JA, up-regulation was found for vegetative storage protein (*VSP1*), JA-ZIM-domain protein (*JAZ10*), MYB50 and arginine decarboxylase (*ADC2*).

**Table 5:** *Arabidopsis thaliana* genes commonly expressed at the time of Vm depolarization (16 h) upon *Pseudomonas syringae* infection.

GO Category	AGI	Short description	FC (P<0.05)
Defense response to bacterium	*At5g13320*	GH3-Like Defense Gene Encodes PBS3 (GDG1)	5.57
	*At3g56400*	WRKY70	4.17
	*At1g70690*	plasmodesmal protein (PDLP5)	4.12
	*At1g74710*	isochorismate synthase (ICS1)	3.14
	*At3g49120*	Class III peroxidase (PERX34).	2.52
	*At4g20260*	plasma membrane polypeptide (ATPCAP1)	−2.02
Response to salicylic acid stimulus	*At4g23170*	Receptor-Like Protein Kinase (CRK9)	2.38
	*At1g21250*	cell wall-associated kinase (WAK1)	2.21
	*At3g52430*	phytoalexin-deficient 4 protein (PAD4)	5.08
Response to fungus	*At2g14560*	unknown protein	7.37
	*At4g12490*	protease inhibitor/seed storage/lipid transfer protein (LTP) family protein	6.80
	*At2g39200*	seven-transmembrane domain proteins specific to plants, homolog of MLO12	2.99
defense response, incompatible interaction	*At5g44420*	ethylene- and jasmonate-responsive plant defensin. (PDF1.2A)	2.56
	*At3g25770*	allene oxide cyclase (AOC2)	2.00
Response to jasmonate stimulus	*At5g24780*	vegetative storage protein 1 (VSP1).	7.41
	*At5g13220*	Jasmonate-Zim-Domain Protein (JAZ10)	7.26
	*At1g57560*	MYB50	4.54
	*At4g34710*	arginine decarboxylase (ADC2)	2.51

Values are expressed as fold change with respect to controls (P<0.05). AGI, Arabidopsis Genome Initiative gene index.

The full list of all other Arabidopsis differentially expressed genes upon *P. syringae* infection is reported in Table S5.

## Discussion

### Dynamic V_m_ depolarization responses in Arabidopsis induced by *S. littoralis*, *M. persicae* and *P. syringae* are different

The primary candidate for intercellular signaling in higher plants is the stimulus-induced change in transmembrane potential (V_m_) [39]. Here we showed that a common event upon biotic stress is plant V_m_ depolarization, which occurs at different times according to the biotroph damage. No matter the nature of the biotroph, this event occurs at the same intensity, which in Arabidopsis corresponds to a V_m_ depolarization of about 40 mV. We used the timing of maximal Vm depolarization as a time point to compare the genome-wide response of *Arabidopsis thaliana* to three known pests: two herbivores, *M. persicae* and *S. littoralis* and a pathogen, *P. syringae*.

Two questions arise from our results: 1) why V_m_ depolarization is a common event? 2) Why V_m_ depolarization occurrs at different times? To our knowledge this is the first report on time-course V_m_ variations upon either *M. persicae* or *P. syringae*, whereas more data are available on *S. littoralis* herbivory-induced V_m_ depolarization. Previous studies have demonstrated that *S. littoralis* herbivory triggers a calcium-induced potassium-dependent V_m_ depolarization in wounded tissues [40] followed by a isotropic depolarizing wave that crosses all plant tissues, from shoot to roots [18], [19], [41]–[46]. This effect was found to be strictly dependent on insect oral secretion, since both single and repeated mechanical wounding were not able to induce such response [1]. The lowering of the plant Vm is seen as a insect's strategy to reduced plant cell responses [47], thus it is conceivable to argue that the same strategy might also be used by both *M. persicae* and *P. syringae*. Elicitors are involved in Vm depolarization [48], and both *M. persicae* and *P. syringae* produce elicitors and effectors during biotic attack [16]. Furthermore, the same V_m_ depolarization value recorded upon all three biotic attacks suggests the occurrence of a V_m_ threshold to be reached for successful herbivory/infection.

The second question, why Vm polarization occurs at different times, appears to be associated with the mode of biotic damage. The fast clipping and consistent plant tissue removal by chewing herbivores evidently induces a “quantitative” response that is proportional to tissue damage; on the other hand, the stylet probing and the phloem feeding operated by the aphid induces a reduced amount of damage, that requires more time for a plant response. Finally, bacterial growth and tissue damage takes time, which appears to be proportional to V_m_ depolarization.

### Different patterns of gene regulation responses are induced by the two insects

Our study shows that the molecular mechanism of gene regulation response to *S. littoralis* and *M. persicae* are principally different. The evidence is that *M. persicae* regulated almost 10-fold higher number of genes than *S. littoralis* did. Moreover, genes responsive to both insects were regulated mostly in opposite direction.

A remarkable and specific response to *S. littoralis* herbivory at the time of V_m_ depolarization was the down-regulation of several small heat-shock proteins (sHSP). sHSPs have a high capacity to bind non-native proteins, probably through hydrophobic interaction, and to stabilize and prevent non-native aggregation [49], [50]. Increasing data suggest a strong correlation between sHSP accumulation and plant tolerance to a wide range of stresses [51]. To our knowledge, this is the first report on sHSP down-regulation upon herbivory. Also, down-regulation of HSPs is quite rare although gene expression of sHSPs was down-regulated by SA in Arabidopsis and in tomato [52], whereas treatment by mitochondrial inhibitors and uncouplers down-regulated HSP, suggesting that mitochondrial functions are essential for sHSP synthesis [53].

With regards regulation of transcription, *S. littoralis* herbivory down-regulated a few genes with a regulatory role in mediating crosstalk between signaling pathways for biotic stress responses like DREB TFs [54] and two bHLH TFs. Most of the remaining TFs were up-regulated, including genes involved in the Arabidopsis circadian clock (*REVEILLE 1*, *LHY1*, *CCA1* and *LHY-CCA1-LIKE5*) [55]–[58] and *AtRL2*, which is expressed in the funiculus of ovules and in embryos and is involved in the control of floral asymmetry [59]. Two zinc finger TF were also up-regulated and one of them, *BBX32*, has a native role in mediating gene repression to maintain dark adaptation [60]. An HD-Zip I TF, *ATHB23*, which is under the control of GA and other activators and is involved in establishing polarity during leaf development [61], was also up-regulated by *S. littoralis* herbivory. Altogether, these data indicate an effect of herbivory on the regulation of the circadian clock and the plant development.

Two members of calcium-transporting ATPase (*ACA12* and *ACA13*), that are dramatically induced upon exposure to pathogens [62], were down-regulated by *S. littoralis* herbivory. Ferritin (FER4) was also down-regulated and in Arabidopsis the absence of ferritin induces higher levels of reactive oxygen species, and increased activity of enzymes involved in their detoxification [63], a condition that is commonly found upon *S. littoralis* herbivory [64].

The response of Arabidopsis to *M. persicae* phloem feeding was clearly distinct from responses to the the chewing herbivore *S. littoralis*. After a few hours from feeding, and at the time of maximal V_m_ depolarization, the aphid was able to suppress a consistent number of genes involved in flavonoid metabolism. UV-exposed plants are damaged to a lesser extent by insect herbivores than non-irradiated plants [65], and UV radiation induces the accumulation of flavonoids in Arabidopsis [66]. In general higher concentration of flavonoids constrain aphid reproduction [67]; however, the generalist *M. persicae* was found to perform much better than the specialist *Brevicoryne brassicae*, when both aphids were feeding on *Brassica oleracea* exposed to UV-radiation [68]. The strong down-regulation of the genes coding for anthocyanidin synthases (also known as leucoanthocyanidin dioxygenases, *At4g22870* and *ANS*) and dihydroflavonol reductase (*DRF*), all involved in flavonoid synthesis [69], was correlated with the down-regulation of two MYB TFs (*MYB75* and *MYB123*). MYB123 controls the biosynthesis of flavonoids in the seed coat of Arabidopsis and MYB5 was recently proposed to be partially redundant with MYB123 in this respect ([70] and references therein). Moreover, the up-regulation of flavonoid transferase (*UF3GT*) induced by *S. littoralis* was in contrast with the down-regulation of this gene after *M. persicae* feeding. These data suggest that *M. persicae* has the ability to suppress plant defenses based on flavonoids.

Fatty acid desaturases (FADs) introduce double bonds into the aliphatic tails of fatty acids and influence plant susceptibility to a wide variety of biotic stresses due in part to their critical role in the biosynthesis of the defense hormone JA [71]. Conversely, SA accumulation is enhanced by transient suppression of FAD [72]. Since aphid resistance has been observed in Arabidopsis and tomato mutants deficient in some FADs [73], the up-regulation of FAD-related genes upon *M. persicae* feeding indicates a possible aphid strategy aimed at lowering plant resistance.

Aphids have not been reported to induce detectable levels of JA, probably because of the induction of SA, which can interact antagonistically with JA signaling [26]. However, recent evidence suggests that SA accumulation may not be required for the repression of JA by whiteflies [74]. Our data show that *M. persicae* induction of several FADs and other genes related to fatty acid biosynthetic process was associated with a JA down-regulation. In fact, most of the genes related to JA biosynthesis (*OPR2*, *OPR3*, *AOC3*, *CYP94B3*, *LOX3*, *LOX4*) and signaling (several JAZs and JRs) were down-regulated, as were 8 out of the 12 members of the JAZ family of plant-specific proteins in Arabidopsis. These data are in agreement with a down-regulation of JA-related genes by phloem feeders [73], [74]. JAZ proteins act as indirect transcriptional repressors of JA-dependent responses and their target to be repressed is MYC2, a key transcriptional regulator of JA signaling [75], [76]. MYC2 is proposed to regulate the transcription of defense-related genes directly or indirectly through secondary TFs that mediate transcription of defense-related genes and accumulation of defense metabolites [76]. The fact that MYC2 positively regulates flavonoid biosynthesis and the down-regulation of MYC2 and several MYC2-dependent secondary TFs (e.g., *CHI*, *ERF1*) by *M. persicae* suggest that the aphid is able suppress plant secondary metabolism at several levels.

*M. persicae* feeding induced up-regulation of three families of early auxin-responsive genes, auxin/indole acetic acid (Aux/IAA), *GH3*, and small auxin-up RNA (SAUR), which are usually specifically induced by auxin within minutes [77]. Interestingly, two genes involved in main auxin biosynthetic pathway in Arabidopsis, YUCCA 2(At4g13260) and TAR2 (At4g24670) were up-regulated after *M. persicae* attack. Therefore, we can assume that up-regulation of auxin responsive genes under *M. persicae* attack might be linked to increased auxin concentration due to activation of auxin biosynthesis through YUCCA-TAA1 pathway [78].

*M. persicae* also induced several classes of expansins. Expansin activity is mainly determined by transcription, which in turn is finely regulated by phytohormones [79], [80] and increases after cell wall acidification [81]. *M. persicae* induction of expansins was correlated also with the up-regulation of GA metabolism. Two GA-regulated proteins (*GASA5* and *GASA6*) showed a strong and opposite regulation. *GASA5* was strongly down-regulated by *M. persicae* and the expression of this gene was also found to be inhibited by heat stress but unaffected by the application of exogenous SA, whereas the expression of NPR1, a key component of the SA-signaling pathway, was down-regulated by *GASA5* overexpression [82]. GASA6, which was up-regulated by *M. persicae*, is thought to be a secreted peptide hormone precursor [83] and has also been characterized as an in vivo sugar marker gene [84]. Recently it was shown that *GASA6* is severely repressed by a zinc-finger protein (*AtTZF1*) over-expression, a regulator connecting sugar, ABA, GA and peptide hormone responses [85]. The finding that several zinc-finger TFs were down-regulated by *M. persicae* correlates with the up-regulation of *GASA6*. The down-regulation of gene coding for α-amilase (*AMY1*) and DELLA (*RGL3*) was in line with the up-regulation of genes involved in GA synthesis.

Two groups of transporters showed opposite regulation. Many secondary metabolites are transported into cells and across the plasma membrane via endogenous membrane transporters [86]. At the time of V_m_ depolarization during *M. persicae* feeding, almost all peptide transporters were down-regulated. The peptide transporter family consists of electrochemical potential-driven transporters that catalyze uptake of their solutes by a cation-solute symport mechanism [87]. *M. persicae* regulated the gene expression of both yellow stripe-like (YSL) proteins and the OPTs. While YSL transporters are involved in metal homeostasis through the translocation of metal-chelates, OPT proteins play a role in plant growth and development being involved in long-distance metal distribution, nitrogen mobilization, heavy metal sequestration, and glutathione transport ([88] and references therein). MATE efflux proteins were also down-regulated by *M. persicae*. In *Nicotiana tabacum* a tonoplast jasmonate-inducible MATE transporter was found to play an important role in the nicotine translocation by acting as a secondary transporter responsible for unloading of alkaloids in the aerial parts and deposition in the vacuoles [89]. Thus, the down-regulation of secondary plant products and the suppression of JA-induced genes might be another strategy of plant defense suppression by *M. persicae*.

Finally, a large number of TFs expressed in response to chitin were down-regulated by *M. persicae*, including several WRKY, MYB and ERF.

Chitin, a polysaccharide composed of β-1-4-linked N-acetyl-d-glucosamine found in the exoskeleton of insects, has been shown or implicated as a signal in plant defense by inducing regulation of different ERF, MYB and WRKY TFs [90]–[93]. Among these TFs, *WRKY53*, *MYB44* and *RRTF1* were down-regulated by *M. persicae*. *WRKT53* has been shown to be required to fully silence SA biosynthesis [94] and in rice its over-expression resulted in enhanced resistance to pathogens [95]. *MYB44* is required for the induction of resistance to *M. persicae* in Arabidopsis and also affects the repression of aphid feeding activities [96], whereas several ERFs, including *RRTF1*, *ERF1* and *ERF13* were up-regulated upon chitooctaose treatment in Arabidopsis [90] or after feeding of the aphids *Macrosiphum euphorbiae* in tomato and *Aphis gossypii* in melon [97].

### Common and specific response of Arabidopsis infected by *P. syringae*

Our results indicates that common responses between *S. littoralis* and *P. syringae* exist. We found that over 190 genes regulated by *S littoralis*, 32% (61 genes) were also regulated by *P. syringae*. Moreover, this common responsive genes were regulated in the same direction.

It is interesting to note that many genes coding for sHSPs regulated by *S. littoralis* herbivory were also down-regulated upon *P. syringae* infection but not by *M. persicae*. Since some HSP, like Hsp70, are essential for mediating virulence effect of virulence effector of pathogenic *P. syringae* (e.g., Hopl1) and play roles in basal resistance to nonpathogenic strains of *P. syringae* [98], [99], we might argue that down-regulation of HSP could be a common strategy for *P. syringae* and *S. littoralis* to suppress plant defense. Another common feature of *S. littoralis* and *P. syringae* was the up-regulation of EF-hand-containing proteins, which are likely to be the key transducers mediating Ca^2+^ action [100]. Calcium signaling has been demonstrated to play an important role in both plant-pathogen [101] and plant-insect interactions [102].

As expected, the specific response of Arabidopsis to *P. syringae* infection was the up-regulation of genes responding to bacterium, including several SA-related genes. GDG1, a member of the GH3-like gene family, was shown to be an important component of SA-mediated defense against *P. syringae* [103], whereas the transcription factor WRKY70 positively regulates basal resistance to *P. syringae* and is involved in SA-signaling pathway [104]. CRK9 was found to be involved in plant responses to biotic stress, like many other Arabidopsis CRKs [105]–[107], whereas *Phytoalexin Deficient4* (PAD4) stimulates production of SA and other processes to limit pathogen growth and has a distinct function in the plant innate immune response [108]. Wall-Associated Kinase (WAK1) is a candidate receptor of oligogalacturonides [109] that triggers defense responses effective against fungal and bacterial pathogens and is induced by SA [110]. Moreover, SA is primarily synthesized from chorismate via isochorismate through the action of isochorismate synthase 1 (ICS1) [111], a gene required for plant defense that was also up-regulated upon *P. syringae* infection. Up-regulation was also found for a plasmodesmal protein (*PDLP5*) that was recently discovered to be essential for conferring enhanced innate immunity against bacterial pathogens in a SA-dependent manner [112].

*P. syringae* also differentially up-regulated genes responding to JA: JAZ10, which is a negative regulator of both JA signaling and disease symptom development [113]; vegetative storage protein1 (*VSP1*), which is a methyl-JA-inducible gene involved in ABA-dependent stomatal closure in Arabidopsis guard cells [114]; *MYB50*, that is regulated by JA [115] and *ADC2*, which induction has been reported for various stresses and various growth regulators including JA [116], [117]. Allene oxide cyclase (AOC2), that catalyzes an essential step in JA biosynthesis [118] and is induced by JA [119] was also up-regulated. The ethylene- and jasmonate-responsive plant defensin PDF1.2A, a known JA-responsive gene [120], was also up-regulated.

### A few genes are commonly regulated by all three biotic stresses

Surprisingly only a few genes were commonly expressed at the time of V_m_ depolarization. In particular, four UGTs were down-regulated in all treatments. UGTs convey the transfer of glycosyl residues from activated nucleotide sugars to a wide range of acceptor molecules such as secondary metabolites, including SA and phytoalexins [121]. Down-regulated UGTs belonged mostly to group D, whose members are associated with disease resistance against some pathogens, and a member of Group L that plays an important role in local and systemic resistance in Arabidopsis [122]. A common up-regulation was found for AtNUDX6, the gene encoding ADP-ribose (Rib)/NADH pyrophosphohydrolase. AtNUDX6 is a modulator of NADH rather than ADP-Rib metabolism and significantly impacts the plant immune response as a positive regulator of NPR1-dependent SA signaling pathways [123]. The TIR domain is found in one of the two large families of homologues of plant disease resistance proteins (R proteins) [124]. The largest class of known resistance proteins includes those that contain a nucleotide binding site and leucine-rich repeat domains (NBS-LRR proteins) [125]. The commonly up-regulated gene *At5g45000* is a TIR-NBS-LRR class domain-containing disease resistance protein, transmembrane receptor involved in signal transduction, defense response and innate immune response [125], [126]. Another commonly up-regulated gene was a heavy-metal-associated domain-containing protein (*At5g26690*) which was found in the category of SA-induced genes positively regulated by a n-butanol-sensitive pathway, implying a cross-talk between SA and phospholipase D pathways [127].

Thus, at the time of V_m_ depolarization a common plant responses to the two herbivores and the pathogen is activation of SA-dependent responses and the triggering of transmembrane receptors, whereas the action of all three biotrophs is the suppression of plant UGTs involved in direct defense.

An unexpected relationship was found for some Arabidopsis genes commonly up-regulated by the herbivore *S. littoralis* and the pathogen *P. syringae* and down- regulated by *M. persicae*. The aphid was found to suppress hydrolase and kinase plant activities, which were activated by the pathogen and the chewing herbivore. Moreover, several oxidative and calcium-related encoding genes as well as some cytochrome P450s (including CYP81F4, involved in the formation of the methoxy group in the indole ring to yield 1-methoxy-indole-3-yl-methyl glucosinolate in Arabidopsis [128]) were only suppressed by the aphid. A remarkable up-regulation was found upon *S. littoralis* herbivory or *P. syringae* infection, and the opposite regulation upon *M. persicae* feeding, for a 2-oxoglutarate (2OG) and Fe(II)-dependent oxygenase (*At4g10500*). In plants, these enzymes catalyze the molecular oxygen reduction at the Fe(II) ion binding residue, where it reacts with 2-oxoglutarate and a specific substrate through the incorporation of one atom of oxygen in each compound and have been involved in plant defense [129].

### Comparison with previous transcriptome analyses

A direct comparison of our data with previous transcriptome analyses indicate similar trend of gene expression in Arabibidopsis upon herbivory by *S. littoralis*, with particular reference to hydrolases (beta-galactosidase, beta-1,3-glucanase, pectinesterase, xyloglucan endo-1,4-beta-D-glucanase, xyloglucan endotransglycosylase), oxido-reductases (monodehydroascorbate reductase, oxidoreductase), transcription factors (ERF/AP2, MYB, WRKY) and other genes induced by biotic stress (NAM, TIR-NBS-LRR class, RPP1, calmodulin, cysteine-rich receptor-like protein kinase). An opposite trend was found for oxophytodienoic acid reductases (OPR1), alcohol dehydrogenase (ADH), arabinogalactan-protein (AGP12), glutathione S-transferase, heat shock protein 70, and a GTP binding protein [28], [130]. However it has to be considered that timing of gene extraction was longer (3–5 h) and falling outside the timing of membrane depolarization. Feeding Arabidopsis with *S. exigua* for longer periods (from 8 to 48 h) showed the same gene expression only for NADP-dependent oxidoreductase and the opposite expression for lipase, 12-oxophytodienoic acid reductase and ERF1, with respect to our results [31], [131].

A comparison with *M. persicae* transcriptome data shows that experiments were mostly performed at longer feeding times, hence beyond the membrane depolarization effect. With respect to our results, after 36 h feeding an opposite gene expression was found for a zinc-binding family protein, a FAD-binding domain-containing protein and for syntaxin [27]. In experiment with feeding times of 48 h [31], lypoxygenase, lipase, coronatine-responsive protein, cellulose synthase, 12-oxophytodienoate reductase, 2-oxoglutarate-dependent dioxygenase, tyrosine decarboxylase, allene oxide cyclase, phenylalanine ammonia-lyase, some WRKY family transcription factors, ethylene response factor 1 (ERF-1), cytochrome B5 family protein 1, amino acid permease and branched-chain amino acid aminotransferase showed the same expression trend; whereas jacalin lectin, flavin-containing monooxygenase, O-methyltransferase, and cytochrome P450 (CYP79B2) showed an opposite trend, with respect to our results. Finally, after 72 h feeding the same gene expression trend was found for copper amine oxidase, fructose-bisphosphate aldolase, glutathione S-transferase, a cytochrome P450 [132], pathogenesis-related protein 1 precursor (PR-1), plant defensin-fusion protein (PDF1.2), superoxide dismutase (Cu-Zn), calmodulin, peroxidase, tyrosine decarboxylase [133] and a sugar transporter family protein [134]. At this timing of aphid feeding, an opposite trend of gene expression with respect to our data was found for coronatine-responsive tyrosine aminotransferase, gibberellin 20-oxidase, heat shock protein 81-1 (HSP81-1), octicosapeptide/Phox/Bem1p (PB1) domain-containing protein, purple acid phosphatase, glycosyl hydrolase family, copper amine oxidase, jacalin lectin family protein, dehydrin xero2 (XERO2), AP2 domain-containing transcription factor, ethylene-responsive element-binding protein 1 (ERF1), respiratory burst oxidase protein D (RbohD), lipoxygenase, cytochrome P450s CYP79B2 and P450 CYP83B1, IAA-amino acid hydrolase 3 and 6, glycosyl hydrolase family 1 [132] glutathione S-transferase, phenylalanine ammonia-lyase, and endotransglycosylase [133].

Finally, a comparison with literature data on Arabidopsis transcriptome after infection by *P. syringae* DC3000 indicates that most of the experiments were carried on at longer times of infection, with respect to the maximum depolarization time observed in our experiments. After 24 h infection, the same gene expression was still found for many genes, including a stress-responsive protein (At1g29395), leucine-rich repeat protein kinases (At1g51800, At1g51850, At1g51860), glutathione S-transferase, DC1 domain-containing protein, cytochrome P450 (CYP71A12), MLO-like protein 12 (MLO12), protein phosphatase 2C, expressed protein (At3g18250), aspartyl protease family protein, transport protein SEC61 beta 1 subunit, chitinase, protease inhibitor/seed storage/lipid transfer proteins (LTP) (At4g12490, At4g12500), protein kinase, zinc finger (CCCH-type) family protein, nodulin family protein and beta-galactosidase [135]. However, after 24 h infection, eight of the 12 JAZ genes are induced during infection in a COR-dependent manner showed an opposite gene expression [113]. Three to four days after infection several genes show the same regulation as observed in our 16 h experiment. These included cell cycle control protein-related, glutathione S-transferase, expressed protein (At1g13340), auxin-responsive GH3 family protein, cation/hydrogen exchanger (CHX17), UDP-glucoronosyl/UDP-glucosyl transferase, disease resistance protein (TIR class), AAA-type ATPase family protein, zinc finger (C3HC4-type RING finger) family protein, cytochrome P450 (CYP71A12), protease inhibitor/seed storage/lipid transfer protein (LTP), cyclic nucleotide-regulated ion channel (CNGC10) (ACBK1), calmodulin-binding protein, avirulence induced gene (AIG1), isochorismate synthase 1 (ICS1), phytoalexin-deficient 4 protein (PAD4), WRKY4, vegetative storage protein 1 (VSP1) [34] and WRKY70 [136]. In comparison to our results, an opposite regulation after 3–4 days on infection was observed for :pectinesterase family protein, O-methyltransferase 1, leucine-rich repeat protein kinase, jacalin lectin family protein, ankyrin repeat family protein, PDF1.2, DC1 domain-containing protein, pectinesterase family protein, chitinase [34], CPR5, JAR1, COI1 and PR1 [136].

## Conclusions

The finding that plant plasma membranes respond with a similar V_m_ depolarization at times depending on the nature of biotic attack allowed us to set a time point for comparative genome-wide analysis among different pests. Our results showed that the aphid *M. persicae* regulates a wider array of Arabidopsis genes with a clear and distinct regulation than the chewing herbivore *S. littoralis*. Despite the different timing of V_m_ depolarization, *S. littoralis* and *P. syringae* share different commonly regulated genes, implying a relationship between V_m_ depolarization and gene expression. Although several commonly expressed genes between the aphid and the pathogen were present, an almost completely opposite regulation was observed, with the aphid suppressing and the pathogen activating plant defense responses.

## Materials and Methods

### Plant, animal and microbial material

*Arabidopsis thaliana* L. (Columbia 0) plants were grown from seed in a plastic pot with sterilized potting soil at 23°C and 60% humidity using daylight fluorescent tubes (120 µE m^−2^ s^−1^) with a photoperiod of 16 h. Every liter of soil contained 15 g of vermiculite and 335 g of soil. Moreover, two fertilizers, Osmocote® and Triabon® (16-8-12-4+TE), were added at a concentration of 1 g l^−1^ soil. All experiments were carried out using 20–22 days-old plants (phase 3) whose leaves turned out to be the most responsive to external stimuli. At least three leaves per plant were used for infestation.

The avirulent strain (Avr) of *Pseudomonas syringae* pv tomato DC3000 (Pst DC3000 AvrRPM1) was cultured at 28°C in NYGB medium (bactopeptone 5 g l^−1^, yeast extract 3 g l^−1^, glycerol 20 g l^−1^, bactoagar 15 g l^−1^) supplemented with rifampicin (100 µg ml^−1^) and kanamycin (25 µg ml^−1^) (all supplied by Sigma, Milan, Italy). Bacterial cultures were centrifuged at 2500 g for 15 min to recover bacteria, which were resuspended in sterile water to a final OD_600_ of 0.2 (equivalent to 1×10^8^ bacteria/ml). Dilution plating was used to confirm the number of bacteria present in the inoculum.

Virus-free *Myzus persicae* aphids were reared on *A. thaliana* plants. They were allowed to grow inside Plexiglas boxes at the temperature of 23°C with a 16 h photoperiod. Only apterous aphids were used in the experiments. Prior to each experiment, at least 20 nymphs per plant were selected for the infestation.

Larvae of *Spodoptera littoralis* (Boisd. 1833) (Lepidoptera, Noctuidae) (supplied as egg clutches by Syngenta, Switzerland), were reared in Petri dishes at 22–24°C with a 14–16 h photoperiod and fed artificially with a diet as detailed elsewhere [1]. Only third instar larvae (at least 5 per plant) were used for herbivory and leaves were collected when 30% fed by larvae.

Plant defense responses induced by herbivores used as controls mechanical damage, which was inferred with either a pattern wheel (to mimic *S. littoralis* herbivore damage) or a microforged glass micropipettes (to mimic aphid stinging). *P. syringae* inoculation used as a control inoculation with MgCl_2_.

### Membrane potentials

Membrane potentials were determined in leaf segments in time course experiments. The transmembrane potential (V_m_) was determined with glass micropipettes as previously described [1], [19], [137]. Based on topographical and temporal determination of V_m_ performed previously [18] the electrode was inserted between 0.5 and 2 mm from the wounded zone, where a significant V_m_ depolarization was found to occur. The results of V_m_ are shown as the average number of at least 50 V_m_ measurements.

### RNA extraction from Arabidopsis leaves upon insect attack and bacterial infection and cDNA synthesis

Having assessed the timing of V_m_ depolarization, after each experiment, leaves were collected and immediately frozen in liquid nitrogen. Samples for the evaluation of *S. littoralis* herbivory were sampled after 2 h from feeding, whereas samples for *M. persicae* phloem feeding were sampled after 5 h from feeding. Samples for the evaluation of Arabidopsis responses to *P. syringae* were sampled after 16 h from infiltration. Four biological replicates were run for each tested biotic attack.

One hundred mg of frozen leaves were ground in liquid nitrogen with mortar and pestle. Total RNA was isolated using the Agilent Plant RNA Isolation Mini Kit (Agilent Technologies, Santa Clara, CA, US) and RNase-Free DNase set (Qiagen, Hilden, Germany). Sample quality and quantity was checked by using the RNA 6000 Nano kit and the Agilent 2100 Bioanalyzer (Agilent Technologies) according to manufacturer's instructions. Quantification of RNA was also confirmed spectrophotometrically by using a NanoDrop ND-1000 (Thermo Fisher Scientific, Waltham, MA, US).

### Gene microarray analyses (including MIAME)

Five hundred nanograms of total RNA from each treated samples, were separately reverse-transcribed into double-strand cDNAs by the Moloney murine leukemia virus reverse transcriptase (MMLV-RT) and amplified for 2 h at 40°C using the Agilent Quick Amp Labelling Kit, two-color (Agilent Technologies). Subsequently, cDNAs were transcribed into antisense cRNA and labeled with either Cy3-CTP or Cy5-CTP fluorescent dyes for 2 h at 40°C following the manufacturer's protocol. Cyanine-labeled cRNAs were purified using RNeasy Minikit (Qiagen, Hilden, Germany). Purity and dye incorporation were assessed with the NanoDrop ND-1000 UV-VIS Spectrophotometer (Thermo Fisher Scientific, Waltham, MA, US) and the Agilent 2100 Bioanalyzer (Agilent Technologies). Then, 825 ng of control Cy3-RNAs and 825 ng of treated Cy5-RNAs were pooled together and hybridized using the Gene Expression Hybridization Kit (Agilent Technologies) onto 4×44 K Arabidopsis (v3) Oligo Microarray (Agilent Technologies), satisfying Minimum Information About a Microarray Experiment (MIAME) requirements [138].

After a 17 h incubation at 65°C and 10 rpm, microarrays were first washed with Gene Expression Wash buffer 1 for 1 min, then with Gene Expression Wash buffer 2 for 1 min, then with 100% acetonitrile for 30 s, and finally washed in the Stabilization and Drying Solution for 30 s.

Microarrays were scanned with the Agilent Microarray G2505B Scanner (with the extended dynamic range (XDR) scan mode to scan the same slide at two different levels and data were extracted and normalized from the resulting images using Agilent Feature Extraction (FE) software (v.9.5.1)

Agilent Arabidopsis (v3) Gene Expression Microarray (44 K) was used for expression profiling treatments. The microarray experiment followed a direct 2×2 factorial two-color design. For each of the treatment combinations, RNA from three independent lines was extracted and used for hybridization. For each RNA samples, four biological replicates were used. This resulted in 12 two-color arrays.

GO enrichment information for the differently expressed probe sets was performed using EasyGO (http://bioinformatics.cau.edu.cn/easygo/category_treeBrowse.html).

The microarray data is MIAME compliant and has been submitted to GEO database with Accession number pending.

### Validation of microarrays

Validation of the microarray analysis was performed by quantitative real time PCR. First strand cDNA synthesis was accomplished with 1.5 µg total RNA and random primers using the High-Capacity cDNA Reverse Transcription Kit (Applied Biosystems, Foster City, CA, US), according to the manufacturer's instructions. Briefly, the reactions were prepared by adding 10 µl total RNA (1.5 µg), 2 µl of 10× RT Buffer, 0.8 µl of 25× dNTPs mix (100 mM), 2 µl 10× RT random primer, 1 µl of Multiscribe™ Reverse Transcriptase and nuclease-free sterile water up to 20 µl. Then the reaction mixtures were subjected to thermal incubation according to the following conditions; 25°C for 10 minutes, 37°C for 2 hours, and 85°C for 5 seconds.

All qPCR experiments were performed on a Stratagene Mx3000P Real-Time System (La Jolla, CA, USA) using SYBR green I with ROX as an internal loading standard. The reaction was performed with 25 µl of mixture consisting of 12.5 µl of 2× Maxima™ SYBR Green/ROX qPCR Master Mix (Fermentas International, Inc, Burlington, ON, Canada), 0.5 µl of cDNA and 100 nM primers (Integrated DNA Technologies, Coralville, IA, US). Controls included non-RT controls (using total RNA without reverse transcription to monitor for genomic DNA contamination) and non-template controls (water template). Specifically, PCR conditions were the following: plant cadmium resistance 1 (*PCR1*, *At1g14880*), HSP20-like chaperones superfamily (*At1g52560*): initial polymerase activation of 10 min at 95°C, and 40 cycles of 30 s at 95°C, 30 sec at 56°C, and 45 sec at 72°C; methionine reductase B8 (*MSRB8*, *At4g21840*), UDP-glycosyltransferase 73B4 (*UGT73B4*, *At2g15490*): initial polymerase activation of 10 min at 95°C, and 40 cycles of 15 s at 95°C, 30 sec at 59°C, and 30 sec at 72°C; germin-like protein (*GR3*, *At5g20630*), flowering promoting factor 1 (*At5g10625*), cytoplasmic glyceraldehyde-3-phosphate dehydrogenase, (*GAPC2*, *At1g13440*), ubiquitin specific protease 6 (*UBP6*, *At1g51710*), β-adaptin (*At4g11380*), elongation factor 1B alpha-subunit 2 (*eEF1Balpha2*, *At5g19510*): initial polymerase activation of 10 min at 95°C, and 40 cycles of 15 s at 95°C, 20 sec at 57°C, and 30 sec at 72°C. Fluorescence was read following each annealing and extension phase. All runs were followed by a melting curve analysis from 55 to 95°C. The linear range of template concentration to threshold cycle value (Ct value) was determined by performing a dilution series using cDNA from three independent RNA extractions analyzed in three technical replicates. All primers were designed using Primer 3 software [139]. Primer efficiencies for all primers pairs were calculated using the standard curve method [140]. Four different reference genes (cytoplasmic glyceraldehyde-3-phosphate dehydrogenase, (*GAPC2*, *At1g13440*), ubiquitin specific protease 6 (*UBP6*, *At1g51710*), β-adaptin (*At4g11380*) and the elongation factor 1B alpha-subunit 2 (*eEF1Balpha2*, *At5g19510*) were used to normalize the results of the real time PCR. The best of the four genes was selected using the Normfinder software [141]; the most stable gene was the elongation factor 1B alpha-subunit 2.

All amplification plots were analyzed with the MX3000P™ software to obtain Ct values. Relative RNA levels were calibrated and normalized with the level of the elongation factor 1B alpha-subunit 2 mRNA.

Primers used for real-time PCR were as follows: Plant cadmium resistance 1 (*PCR1*, *At1g14880*) forward primer 5′-GATCGAGGATCCAAATCGTG-3′, reverse primer 5′-TGTTGGGTCAAAGCACAGAG-3′; HSP20-like chaperones superfamily (*At1g52560*) forward primer 5′-GCTCACCTGAGGAAGACGAG-3′, reverse primer 5′-TCCGCCTTAATGTCCTCAAC-3′; methionine reductase B8 (*MSRB8*, *At4g21840*) forward primer 5′-CTAAGTTCGACTCCGGTTGC-3′, reverse primer 5′-TGGCCTAGATGTCCATCACA-3′; UDP-glycosyltransferase 73B4 (*UGT73B4*, *At2g15490*) forward primer 5′-TTGGTTGCCTAAAGGGTTTG-3′, reverse primer 5′-TCCAAAGTCGAGTTCCATCC-3′; Germin-like protein (*GR3*, *At5g20630*) forward primer 5′-CATCCTGGTGCTTCTGAGGT-3′, reverse primer 5′-GGGCCTTTCCCAGAGTTTAG-3′; flowering promoting factor 1 (*At5g10625*), forward primer 5′-CTAGTGGAGAACCCGAACCA-3′, reverse primer 5′-TGTTCGAGCGACGAGTAAGA-3′; elongation factor 1B alpha-subunit 2 (*eEF1Balpha2, At5g19510*) forward primer 5′-ACTTGTACCAGTTGGTTATGGG-3′, reverse primer 5′-CTGGATGTACTCGTTGTTAGGC-3′; ubiquitin specific protease 6 (*UBP6*, *At1g51710*) forward primer 5′-GAAAGTGGATTACCCGCTG-3′, reverse primer 5′-CTCTAAGTTTCTGGCGAGGAG-3′; cytoplasmic glyceraldehyde-3-phosphate dehydrogenase (*GAPC2, At1g13440*) forward primer 5′-TCAGGAACCCTGAGGACATC-3′, reverse primer 5′- CGTTGACACCAACAACGAAC-3′; β-adaptin (*At4g11380*) forward primer 5′- CACGAGCGTCGAATCAACTA-3′, reverse primer 5′-ATCTCGGGAGTGGGAGTTTT-3′.

Validation of microarray gene expression is shown in Table S6.

### Statistical analyses

For V_m_ measurements, the obtained data were treated by using the stem-and-leaf function of Systat 10 in order to calculate the lower and upper hinge from the Gaussian distribution of values. The data were then filtered and the mean value was calculated along with the SE. Paired t test and Bonferroni adjusted probability were used to assess the difference between treatments and the control.

Processing and statistical analysis of the microarray data were done in R using Bioconductor package limma [142]. The raw microarray data are subjected to background subtraction and loess normalized. Agilent control probes were filtered out. The linear models implemented in limma were used for finding differentially expressed genes. Comparisons were made for each of the treatment and genotype combinations. To reduce the complexity of the analysis, technical replicates were treated as biological replicates. Benjamini and Hochberg (BH) multiple testing correction was applied. We consider genes with both BH adjusted p-value<0.05 and fold changes >2 as differentially expressed genes.

## Supporting Information

Table S1 ***Arabidopsis thaliana*** **genes commonly expressed at the time of Vm depolarization upon** ***Myzus persicae*** **(5 h) herbivory and** ***Pseudomonas syringae*** **(16 h) infection.**
(DOCX)

Table S2 **Other differentially regulated genes upon** ***Spodoptera littoralis*** **herbivory on** ***Arabidopsis thaliana*** **leaves.**
(DOCX)

Table S3 ***Arabidopsis thaliana*** **genes commonly expressed at the time of Vm depolarization (5 h) upon** ***Myzus persicae*** **herbivory.**
(DOCX)

Table S4 **Other genes differentially expressed upon** ***Myzus persicae*** **phloem feeding on** ***Arabidopsis thaliana*** **leaves.**
(XLSX)

Table S5 **Other differentially expressed genes upon infection by** ***Pseudomonas syringae*** **on** ***Arabidopsis thaliana*** **leaves.**
(XLSX)

Table S6 **Validation of microarray data.**
(DOCX)
