# Supplementary material for: Dynamics of Membrane Potential Variation and Gene Expression Induced by Spodoptera littoralis, Myzus persicae, and Pseudomonas syringae in Arabidopsis
Source: PLoS One. 2012 Oct 30;7(10):e46673. doi: 10.1371/journal.pone.0046673 (PMC3484130; doi:10.1371/journal.pone.0046673)
Supplement: Table S1 — Arabidopsis thaliana genes commonly expressed at the time of Vm depolarization upon Myzus persicae (5 h) herbivory and Pseudomonas syringae (16 h) infection. (DOCX) [file pone.0046673.s001.docx]

**Supporting Table S1.** *Arabidopsis thaliana* genes commonly expressed at the time of Vm depolarization upon *Myzus persicae* (5 h) herbivory and *Pseudomonas syringae* (16 h) infection. Values are expressed as fold change with respect to controls (P<0.05). AGI, Arabidopsis Genome Initiative gene index.

| **GO category** | **AGI** | **Short description** | ***P. syringae*** | ***M. persicae*** |
| --- | --- | --- | --- | --- |
| **Multi-organism process** | | |  |  |
| Response to bacterium | At1g02450 | modulates PR gene expression (NIMIN-1) | 13.27 | 8.12 |
|  | At3g22231 | Pathogen and Circadian Controlled 1 (PCC1) | 6.58 | 3.16 |
|  | At2g19190 | FLG22-INDUCED RECEPTOR-LIKE KINASE 1 (FRK1) | 12.95 | -9.01 |
|  | At1g02930 | glutathione transferase (GST1) | 2.18 | -9.71 |
|  | At1g02920 | glutathione transferase (GST11) | 3.23 | -2.79 |
|  | At4g14400 | ACCELERATED CELL DEATH 6 (ACD6) | 2.39 | 2.27 |
|  | At5g01600 | ferretin protein (FER1) | 2.03 | -6.80 |
| Response to other organism | At3g11480 | methyltransferase (BSMT1) | 19.58 | -16.55 |
|  | At4g37150 | methyl jasmonate esterase (MES9) | 3.14 | -6.46 |
|  | At1g19640 | jasmonic acid carboxyl methyltransferase (JMT) | 2.79 | -2.13 |
|  | At4g39030 | enhanced disease susceptibility 5 (EDS5). | 5.37 | -2.87 |
|  | At2g15480 | UDP-glucosyl transferase (UGT73B5) | -3.69 | -4.15 |
|  | At2g43510 | trypsin inhibitor protein 1 (ATTI1) | 2.64 | -2.71 |
|  | At1g75040 | Thaumatin-like pathogenesis-related gene 5 (PR5) | 9.02 | -2.55 |
|  | At1g45145 | cytosolic thioredoxin (ATTRX5) | 5.08 | -2.47 |
|  | At2g14560 | late upregulated in response to *Hyaloperonospora parasitica* (LURP1) | 8.72 | 2.18 |
|  | At1g11330 | S-locus lectin protein kinase family protein | 3.51 | 2.41 |
|  | At5g40020 | pathogenesis-related thaumatin family protein | -2.13 | 2.71 |
| **Response to stimulus** | | |  |  |
| response to stress | At1g52890 | NAC transcription factor (NAC019) | 2.09 | -5.86 |
|  | At1g78410 | VQ motif | 4.54 | -2.84 |
|  | At3g15950 | Similar to TSK-associating protein 1 (TSA1) | 2.60 | -5.42 |
|  | At3g16460 | Mannose-binding lectin superfamily protein (JAL34) | 4.33 | -3.07 |
|  | At5g13930 | chalcone synthase (CHS) | 2.12 | -4.86 |
| Defense response | At1g66100 | pathogenesis-related protein (PR13) | 2.86 | 2.25 |
|  | At3g23120 | Receptor-Like Protein (RLP38) | 5.93 | 2.17 |
|  | At3g25010 | Receptor-Like Protein (RLP41) | 10.81 | -3.11 |
|  | At3g28890 | Receptor Like Protein (RLP43) | 4.31 | -4.92 |
|  | At4g23140 | receptor-like protein kinase (CRK6) | 5.13 | -9.48 |
|  | At4g23150 | receptor-like protein kinase (CRK7) | 9.34 | -7.80 |
|  | At4g23310 | Receptor-Like Protein Kinase (CRK23) | 7.64 | -8.51 |
|  | At4g04490 | receptor-like protein kinase (CRK36) | 4.25 | -4.96 |
|  | At4g18250 | receptor serine/threonine kinase, putative | 17.72 | -5.44 |
|  | At5g01550 | lectin receptor kinase subfamily A4 (LECRKA4.2) | 6.19 | -2.72 |
| Response to stimulus | At1g03850 | glutaredoxin (GRXS13) | 4.06 | 2.11 |
|  | At1g28480 | glutaredoxin (GRX480) | 8.20 | -2.23 |
|  | At5g54610 | ankyrin repeat protein family, (ANK) | 4.40 | 3.36 |
|  | At1g34180 | NAC domain containing protein (NAC016) | 3.27 | -2.61 |
|  | At3g12830 | auxin-responsive family protein | 2.94 | -3.53 |
|  | At3g63010 | gibberellin receptor (GID1B) | 2.26 | -5.43 |
|  | At5g13220 | jasmonate-ZIM-domain protein (JAZ10) | 7.02 | -7.24 |
|  | At2g47000 | ABC transporter (ABCB4). | -2.14 | -2.51 |
|  | **Other Highly regulated genes** | | | |
| Transferase and Transporter activity | At1g70260 | nodulin MtN21 family protein | -2.04 | -9.67 |
|  | At1g61800 | glucose6-Phosphate/phosphate transporter (GPT2) | 4.92 | -15.27 |
|  | At4g35180 | Lys/His transporter (LHT7) | 19.78 | -2.37 |
|  | At3g21080 | ABC transporter-related | 12.74 | -3.21 |
|  | At1g76790 | indole glucosinolate o-methyltransferase (IGMT5) | 3.83 | -8.39 |
|  | At5g07010 | sulfotransferase (ST2A) | 7.11 | -7.46 |
|  | At5g46050 | peptide transporter (PTR3) | 4.72 | -6.38 |
| Hydrolase activity | At5g11920 | fructan exohydrolase (CWINV6) | 29.74 | -5.33 |
|  | At2g43570 | Chitinase (CHI) | 12.87 | -8.06 |
|  | At1g54010 | GDSL-like Lipase/Acylhydrolase superfamily protein | 10.81 | -7.17 |
| Kinase activity | At5g67080 | MAPKKK19 | 2.27 | -9.30 |
|  | At3g25882 | NPR1/NIM1-interacting (NIMIN-2) | 9.14 | 2.31 |
| Transcription factors | At5g22570 | WRKY38 | 12.21 | 2.47 |
|  | At2g40740 | WRKY55 | 8.42 | -3.88 |
| Other response to biotic stress | At1g14120 | 2-oxoglutarate-dependent dioxygenase, putative | 4.12 | -23.79 |
|  | At3g49340 | cysteine proteinase, putative | 3.21 | -12.11 |
|  | At4g21840 | methionine sulfoxide reductase (MSRB8) | 36.61 | -6.38 |
|  | At3g45130 | lanosterol synthase (LAS1) | 32.66 | -2.50 |
|  | At2g06255 | early flowering elf4-like 3 (ELF4-L3) | 5.93 | -3.25 |
|  | At3g55970 | jasmonate-regulated gene (JRG21), | 8.68 | -2.76 |
|  | At4g20000 | VQ motif-containing protein | 8.77 | -2.93 |
|  | At1g65690 | Late embryogenesis abundant (LEA) | 2.28 | -6.21 |
|  | At4g10290 | RmlC-like cupins superfamily protein | 11.99 | -5.30 |
|  | At5g10625 | unknown protein , | 2.61 | -29.44 |
|  | At3g13950 | unknown protein | 12.90 | -6.96 |
|  | At1g11850 | unknown protein | 2.64 | 6.86 |

| **Other genes** | | | | |
| --- | --- | --- | --- | --- |
| **Transferase and Transporter activity** | At1g05020 | epsin N-terminal homology (ENTH) domain-containing protein / clathrin assembly protein-related | 3.23 | 2.37 |
|  | At1g14070 | Xyloglucan fucosyltransferase family | -2.53 | -5.15 |
|  | At1g21110 | O-methyltransferase, putative | 4.18 | -3.32 |
|  | At2g03590 | allantoin transporter | -2.07 | -2.85 |
|  | At2g34940 | vacuolar sorting receptor, putative; functions in: calcium ion binding; involved in: protein targeting to vacuole | 4.49 | 3.04 |
|  | At2g40840 | 4-alpha-glucanotransferase activity, transglucosidase and amylomaltase activity, binds to heteroglycans and utilizes glucose, mannose and xylose as acceptors, preferentially active of β-maltose | -2.31 | -2.47 |
|  | At3g03480 | acetyl CoA:(Z)-3-hexen-1-ol acetyltransferase | 4.64 | -3.87 |
|  | At3g13090 | multidrug resistance-associated protein 8 . ATPase activity. | 2.23 | -2.11 |
|  | At3g44860 | farnesoic acid carboxyl-O-methyltransferase | 2.90 | -3.74 |
|  | At3g44870 | S-adenosyl-L-methionine:carboxyl methyltransferase family protein | 3.16 | -3.54 |
|  |  |  |  |  |
|  |  |  |  |  |
|  | At5g23660 | Nodulin, homolog of the Medicago MTN3 | 3.11 | -2.69 |
|  | At5g60800 | heavy-metal-associated domain-containing protein | 4.53 | 3.40 |
| **Hydrolase activity** | At3g57520 | seed imbibition 2 , hydrolase activity, hydrolyzing O-glycosyl compounds | 2.64 | -3.23 |
|  | At4g17480 | palmitoyl protein thioesterase family protein | 2.71 | -2.09 |
|  | At2g43620 | chitinase, putative | 5.93 | -2.32 |
|  | At1g62770 | invertase/pectin methylesterase inhibitor family protein | -2.80 | -2.48 |
|  | At2g03980 | GDSL-motif lipase/hydrolase family protein | 2.38 | -2.60 |
|  | At1g51780 | IAA-amino acid conjugate hydrolase subfamily and conjugates | 2.17 | -2.96 |
|  | At5g47330 | palmitoyl protein thioesterase family protein | -2.57 | -6.61 |
|  | At5g22860 | serine carboxypeptidase S28 family protein | -2.01 | -2.21 |
|  | At1g13700 | glucosamine/galactosamine-6-phosphate isomerase family protein | -2.27 | 2.19 |
|  | At5g64000 | 3'(2'),5'-bisphosphate nucleotidase | 4.51 | -4.08 |
|  | At3g48080 | lipase class 3 family protein / disease resistance protein-related | 5.51 | 2.31 |
|  | At3g28540 | ATPase family protein, AAA-type | 2.08 | -2.90 |
| **Kinase activity** | At1g51790 | kinase activity | 3.56 | 2.13 |
|  | At1g16260 | protein kinase family protein | 2.15 | 2.02 |
|  | At5g67080 | MEKK subfamily | 2.27 | -9.30 |
|  | At5g59670 | leucine-rich repeat protein kinase, putative | 3.19 | 2.91 |
|  | At5g01850 | protein kinase, putative | 2.99 | -2.08 |
|  | At4g11890 | protein kinase family protein | 6.61 | -2.46 |
| **Transcription factors** | At2g43000 | NAC domain containing protein 42 | 2.28 | -3.52 |
|  | At4g17980 | NAC domain containing protein 71 | -2.50 | -3.67 |
|  | At3g15270 | squamosa-promoter binding protein-like gene family, a novel gene family encoding DNA binding proteins and putative transcription factors | -2.37 | -2.17 |
|  | At2g40200 | basic helix-loop-helix (bHLH) family protein; functions in: transcription factor activity | 4.31 | -2.45 |
|  | At5g24110 | WRKY Transcription Factor; Group III | 5.12 | -4.90 |
| **Response to biotic stress** | At3g22235 | unknown protein, best Arabidopsis thaliana protein match is: PCC1 | 6.29 | 5.80 |
|  | At5g48657 | defense protein-related | 4.25 | -4.10 |
|  | At5g28237 | tryptophan synthase, beta subunit, putative | 2.98 | -5.54 |
|  | At5g44575 | unknown protein | 2.46 | -6.44 |
|  | At1g58225 | unknown protein | 2.54 | -7.29 |
|  | At3g24954 | leucine-rich repeat family protein | 2.02 | -7.52 |
|  | At3g28290 | Integrin similar, Localized to the cytoplasm and plasma membrane | 2.25 | -4.46 |
|  | At1g24145 | unknown protein | 2.95 | -3.53 |
|  | At1g21310 | Extensin | 2.72 | -2.54 |
|  | At1g23730 | beta carbonic anhydrase | -2.18 | -2.46 |
|  | At4g05040 | ankyrin repeat family protein | 2.05 | 2.06 |
|  | At2g41410 | calmodulin, putative | 2.01 | -2.67 |
|  | At1g13830 | beta-1,3-glucanase-related | 4.41 | -6.00 |
|  | At2g38240 | 2OG-Fe(II) oxygenase family protein with oxidoreductase activity | 5.84 | -4.66 |
|  | At5g25820 | exostosin family protein | 2.75 | -2.51 |
|  | At3g26440 | unknown protein | 3.70 | -2.24 |
|  | At4g38560 | unknown protein | 5.23 | -5.20 |
|  | At5g25250 | unknown protein | 7.35 | -2.75 |
|  | At1g68600 | unknown protein | -2.39 | -4.70 |
|  | At3g62780 | C2 domain-containing protein | 2.71 | 2.30 |
|  | At3g51400 | unknown protein | -2.11 | 2.44 |
|  | At3g51440 | strictosidine synthase family protein | 2.40 | -2.22 |
|  | At1g29290 | unknown protein | 2.10 | -3.84 |
|  | At1g63370 | flavin-containing monooxygenase family protein / FMO family protein | -2.48 | -2.58 |
|  | At4g00700 | C2 domain-containing protein | 5.92 | -5.62 |
|  | At2g30830 | 2-oxoglutarate-dependent dioxygenase similar with 1-aminocyclopropane-1-carboxylate oxidase activity | 4.89 | -5.77 |
|  | At5g26280 | meprin and TRAF homology domain-containing protein / MATH domain-containing protein | 2.42 | 4.11 |
|  | At4g33790 | alcohol-forming fatty acyl-CoA reductase, involved in cuticular wax biosynthesis | -4.14 | 5.33 |
|  | At4g30640 | F-box family protein | 4.87 | 2.20 |
|  | At4g15440 | hydroperoxide lyase. Also a member of the CYP74B cytochrome p450 family. | 2.42 | -3.46 |
|  | At5g38900 | DSBA oxidoreductase family protein | 2.42 | -3.40 |
|  | At5g67340 | armadillo/beta-catenin repeat family protein / U-box domain-containing protein | 2.89 | -2.43 |
|  | At2g28630 | 3-ketoacyl-CoA synthase family involved in the biosynthesis of VLCFA (very long chain fatty acids) | -2.47 | 5.47 |
|  | At5g51380 | F-box family protein (.9) | 2.36 | 2.24 |
|  | At2g42350 | zinc finger (C3HC4-type RING finger) family protein | 5.65 | -6.12 |
|  | At3g22240 | unknown protein | 4.93 | 3.95 |
|  | At5g56880 | unknown protein | 3.16 | -6.70 |
|  | At5g62150 | peptidoglycan-binding LysM domain-containing protein | 3.83 | -2.19 |
|  | At4g25433 | peptidoglycan-binding LysM domain-containing protein | 2.43 | -2.63 |
|  | At3g26500 | Plant Intracellular Ras-group-related LRRs (Leucine rich repeat proteins) | 2.85 | -4.97 |
|  | At5g55170 | small ubiquitin-like modifier (SUMO) polypeptide that becomes covalently attached to various intracellular protein targets, much like ubiquitination, leading to post-translational modification of those targets | 3.70 | 2.24 |
|  | At1g78190 | unknown protein | 2.15 | -2.54 |
|  | At3g04000 | short-chain dehydrogenase/reductase (SDR) family protein | 3.72 | -3.73 |
|  | At4g02360 | unknown protein | 2.21 | -6.63 |
|  | At1g26420 | FAD-binding domain-containing protein | 6.21 | -6.66 |
|  | At5g16170 | unknown protein | 4.23 | 3.11 |
|  | At1g23850 | unknown protein | 3.24 | -3.57 |
|  | At4g27860 | integral membrane family protein | 3.30 | -4.74 |
|  | At2g38500 | unknown protein | 3.25 | -3.47 |
|  | At2g47950 | unknown protein | 2.43 | -3.59 |
|  | At1g73325 | trypsin and protease inhibitor family protein / Kunitz family protein | 4.65 | -2.78 |
