# Supplementary material for: Dynamics of Membrane Potential Variation and Gene Expression Induced by Spodoptera littoralis, Myzus persicae, and Pseudomonas syringae in Arabidopsis
Source: PLoS One. 2012 Oct 30;7(10):e46673. doi: 10.1371/journal.pone.0046673 (PMC3484130; doi:10.1371/journal.pone.0046673)
Supplement: Table S2 — Other differentially regulated genes upon Spodoptera littoralis herbivory on Arabidopsis thaliana leaves. (DOCX) [file pone.0046673.s002.docx]

**Supporting Table S2.** Other differentially regulated genes upon *Spodoptera littoralis* herbivory on *Arabidopsis thaliana* leaves.

| **GO Category** | **Gene model** | **Short description** | **FC (P<0.05)** |
| --- | --- | --- | --- |
| Response to high light intensity | *At5g12030* | 17.7 kDa class II heat shock protein 17.6A (HSP17.7-CII) | -22.62 |
|  | *At1g52560* | HSP21 (HEAT SHOCK PROTEIN 21) | -36.21 |
|  | *At2g32120* | HSP70 (heat shock protein 70) | -3.33 |
|  | *At5g59680* | light repressible receptor protein kinase | 6.16 |
| Response to heat | *At1g53540* | 17.6 kDa class I heat shock protein (HSP17.6) | -18.65 |
|  | *At1g07400* | 17.6 kDa class I heat shock protein (HSP17.6A-CI) | -2.60 |
|  | *At2g29500* | 17.6 kDa class I small heat shock protein (HSP17.6B-CI) | -4.71 |
|  | *At1g53540* | 17.6 kDa class I small heat shock protein (HSP17.6C-CI) | -18.65 |
|  | *At5g12020* | 17.6 kDa class II heat shock protein (HSP17.6II) | -10.05 |
|  | *At5g51440* | 23.5 kDa mitochondrial small heat shock protein (HSP23.5-M) | -3.89 |
|  | *At4g25200* | 23.6 kDa mitochondrial small heat shock protein (HSP23.6-M) | -23.74 |
|  | *At1g52560* | 26.5 kDa class I small heat shock protein-like (HSP26.5-P) | -36.21 |
|  | *At1g09080* | BIP (LUMINAL BINDING PROTEIN), ATP binding | 6.05 |
|  | *At1g16030* | HSP70 (heat shock protein 70), | -6.24 |
|  | *At1g74310* | heat shock protein 101 (HSP101) | -3.25 |
| Other abiotic responses | *At1g77760* | nitrate reductase 1 (NR1) | 5.20 |
|  | *At3g59220* | pirin, cupin-domain containing protein | -2.98 |
|  | *At4g25260* | invertase/pectin methylesterase inhibitor family protein / DC 1.2 homolog (FL5-2I22) | -2.02 |
|  | *At5g65080* | MADS-box domain protein. | -6.00 |
| Response to biotic stimulus | *At1g19320* | Pathogenesis-related protein 5 precursor (PR-5) | -2.51 |
|  | *At1g76680* | 12-oxophytodienoic acid reductases (OPR1) | -2.18 |
|  | *At1g76690* | 12-oxophytodienoic acid reductases (OPR2). | -2.43 |
|  | *At2g04450* | Nudix hydrolase 6 (NUDT6) | 3.28 |
|  | *At2g15490* | UDP-glucoronosyl/UDP-glucosyl transferase family protein | -6.81 |
|  | *At3g28740* | cytochrome p450 family | -4.12 |
|  | *At3g50480* | broad-spectrum mildew resistance RPW8 family protein | 2.16 |
|  | *At3g57240* | beta-1,3-glucanase (BG3) | 3.20 |
|  | *At3g57260* | beta 1,3-glucanase | 3.94 |
|  | *At4g34131* | UDP-glucoronosyl/UDP-glucosyl transferase family protein | -2.53 |
|  | *At4g34135* | UDP-glucoronosyl/UDP-glucosyl transferase family protein | -2.82 |
|  | *At1g05680* | UDP-glucoronosyl/UDP-glucosyl transferase family protein | -4.21 |
|  | *At2g36750* | UDP-glucoronosyl/UDP-glucosyl transferase family protein | -6.66 |
|  | *At5g59590* | UDP-glucoronosyl/UDP-glucosyl transferase family protein | -2.77 |
|  | *At5g24530* | oxidoreductase, 2OG-Fe(II) oxygenase family protein | 2.71 |
|  | *At5g47220* | ERF (ethylene response factor) subfamily B-3 of ERF/AP2 transcription factor family (ERF-2) | 3.07 |
|  | *At5g53550* | Metal-nicotianamine transporter YSL3 (YSL3) | 2.31 |
|  | *At1g43910* | AAA-type ATPase family protein | 2.90 |
|  | *At3g55090* | ABC transporter family protein ATP-binding cassette-sub-family G-member 2 | 3.81 |
|  | *At3g13520* | arabinogalactan-protein (AGP12) | -2.54 |
|  | *At2g25130* | armadillo/beta-catenin repeat family protein | -3.17 |
| response to cadmium ion and inorganic ions |  |  |  |
|  | *At1g77120.* | alcohol dehydrogenase (ADH) | -2.26 |
|  | *At5g24380* | metal-phytosiderophore/metal-nicotianamine transporter ZmYS1 | 2.94 |
|  | *At5g17330* | glutamate decarboxylase 1 (GAD 1) | 4.12 |
| Responses to jasmonic acid | *At1g66370* | myb family transcription factor (MYB113) | 4.30 |
|  | *At3g09940* | monodehydroascorbate reductase, cytoplasmic isoform 1 | 3.29 |
| Responses to salicylic acid | *At1g05560* | UDP-glucose transferase (UGT75B2) | -2.39 |
| Other stress responses | *At1g12950* | MATE efflux family protein | -2.23 |
|  | *At1g17170* | glutathione S-transferase, putative | -5.08 |
|  | *At2g29480* | glutathione S-transferase, putative | -4.38 |
|  | *At2g29490* | glutathione S-transferase, putative | -3.45 |
|  | *At2g32680* | disease resistance family protein / LRR family protein | 4.55 |
|  | *At3g01080* | WRKY Transcription Factor; Group I | 4.56 |
|  | *At3g23240* | ERF (ethylene response factor) subfamily B-3 of ERF/AP2 transcription factor family (ERF1). | 2.44 |
|  | *At3g44400* | disease resistance protein (TIR-NBS-LRR class), RPP1 (Recognition of Peronospora Parasitica 1) | 2.18 |
|  | *At3g45860* | Cysteine-rich receptor-like protein kinase 4 precursor (CRK4) | 3.47 |
|  | *At4g04220* | disease resistance family protein / LRR family protein | 2.04 |
|  | *At5g41750* | disease resistance protein (TIR-NBS-LRR class), | 2.63 |
|  | *At1g10340* | ankyrin repeat family protein | 2.86 |
|  | *At5g54710* | ankyrin repeat family protein | 2.75 |
|  | *At4g11000* | ankyrin repeat family protein | 3.47 |
|  | *At4g03450* | ankyrin repeat family protein | 3.23 |
|  | *At3g47480* | calcium-binding EF hand family protein | 2.65 |
|  | *At5g54130* | calcium-binding EF hand family protein | 2.58 |
|  | *At5g39670* | calcium-binding EF hand family protein | 2.45 |
|  | *At5g57010* | calmodulin-binding family protein | -2.34 |
|  | *At5g26920* | calmodulin-binding protein similar to calmodulin-binding protein | 2.08 |
|  | *At3g50770* | calmodulin-related protein, putative | 2.83 |
|  | *At2g47560* | zinc finger (C3HC4-type RING finger) family protein | 3.13 |
|  | *At5g63780* | zinc finger (C3HC4-type RING finger) family protein | -2.41 |
|  | *At3g30460* | zinc finger (C3HC4-type RING finger) family protein | -2.35 |
|  | *At5g67110* | basic helix-loop-helix (bHLH) family protein | -2.94 |
|  | *At5g01900* | WRKY family transcription factor | 3.31 |
| Multi-organism process | *At1g65790* | S-receptor transmembrane protein kinase serine/threonine kinase | 3.65 |
| Secondary metabolic process | *At1g16410* | member of CYP79F | 2.03 |
|  | *At3g19010* | oxidoreductase, 2OG-Fe(II) oxygenase family protein | 2.01 |
|  | *At4g10500* | oxidoreductase, 2OG-Fe(II) oxygenase family protein | 6.41 |
| Other regulated genes | *At1g14880* | expressed protein | 7.39 |
|  | *At1g13470* | expressed protein | 6.16 |
|  | *At4g25110* | latex-abundant family protein (AMC2) / caspase family protein | 5.59 |
|  | *At4g36850* | PQ-loop repeat family protein / transmembrane family protein | 5.22 |
|  | *At5g55460* | protease inhibitor/seed storage/lipid transfer protein (LTP) family protein | 4.87 |
|  | *At5g22380* | no apical meristem (NAM) family protein | 4.77 |
|  | *At2g15020* | expressed protein | 4.71 |
|  | *At5g56870* | beta-galactosidase, putative | 4.20 |
|  | *At1g59590* | expressed protein | 4.10 |
|  | *At4g29690* | type I phosphodiesterase/nucleotide pyrophosphatase family protein | 4.06 |
|  | *At5g54060* | UDP-glucoronosyl and UDP-glucosyl transferase | 3.98 |
|  | *At3g26210* | cytochrome P450 71B23, putative (CYP71B23) | 3.86 |
|  | *At5g26690* | heavy-metal-associated domain-containing protein | 3.74 |
|  | *At3g57460* | expressed protein | 3.67 |
|  | *At1g35710* | leucine-rich repeat transmembrane protein kinase, | 3.51 |
|  | *At4g37410* | cytochrome P450, putative | 3.42 |
|  | *At3g49620* | 2-oxoacid-dependent oxidase, putative (DIN11) | 3.27 |
|  | *At5g24200* | expressed protein | 3.14 |
|  | *At3g01290* | band 7 family protein | 3.05 |
|  | *At3g48640* | expressed protein | 3.00 |
|  | *At1g67000* | protein kinase family protein | 2.97 |
|  | *At1g23840* | expressed protein | 2.96 |
|  | *At2g47130* | short-chain dehydrogenase/reductase (SDR) family protein | 2.96 |
|  | *At5g65090* | endonuclease/exonuclease/phosphatase family protein | 2.91 |
|  | *At4g04810* | methionine sulfoxide reductase domain-containing protein | 2.89 |
|  | *At5g22545* | expressed protein | 2.84 |
|  | *At5g04950* | nicotianamine synthase, putative | 2.82 |
|  | *At4g21850* | methionine sulfoxide reductase domain-containing protein. | 2.81 |
|  | *At3g48360* | speckle-type POZ protein-related | 2.77 |
|  | *At4g29700* | type I phosphodiesterase/nucleotide pyrophosphatase family protein | 2.62 |
|  | *At5g10760* | aspartyl protease family protein | 2.61 |
|  | *At5g20790* | expressed protein | 2.61 |
|  | *At1g65490* | expressed protein | 2.52 |
|  | *At4g32480* | expressed protein | 2.52 |
|  | *At1g24140* | matrixin family protein | 2.51 |
|  | *At1g64500* | glutaredoxin family protein | 2.39 |
|  | *At1g53830* | pectinesterase 2 (PME2/ PE 2) | 2.36 |
|  | *At3g62040* | haloacid dehalogenase-like hydrolase family protein | 2.35 |
|  | *At2g23680* | stress-responsive protein, putative | 2.34 |
|  | *At5g38130* | transferase family protein similar to anthranilate N-hydroxycinnamoyl/benzoyltransferase, | 2.29 |
|  | *At3g26230* | cytochrome P450 family protein | 2.28 |
|  | *At3g61280* | expressed protein | 2.28 |
|  | *At1g48260* | CBL-interacting protein kinase 17 (CIPK17) | 2.26 |
|  | *At5g45000* | Toll-Interleukin-Resistance (TIR) domain-containing protein | 2.26 |
|  | *At1g23870* | glycosyl transferase family 20 protein / trehalose-phosphatase family protein | 2.21 |
|  | *At2g41150.2* | expressed protein | 2.17 |
|  | *At1g66880* | serine/threonine protein kinase family protein | 2.14 |
|  | *At1g17240* | leucine-rich repeat family protein | 2.13 |
|  | *At5g57550* | xyloglucan:xyloglucosyl transferase / xyloglucan endotransglycosylase / endo-xyloglucan transferase (XTR3) | 2.13 |
|  | *At4g24350* | phosphorylase family protein contains Pfam PF01048: Phosphorylase family [At4g24350.1] | 2.12 |
|  | *At1g01390* | UDP-glucoronosyl/UDP-glucosyl transferase family protein | 2.11 |
|  | *At2g41250* | haloacid dehalogenase-like hydrolase family protein | 2.07 |
|  | *At4g37540* | LOB domain protein 39 / lateral organ boundaries domain protein 39 (LBD39) | 2.07 |
|  | *At5g15950* | adenosylmethionine decarboxylase family protein | 2.06 |
|  | *At2g27310* | F-box family protein | 2.05 |
|  | *At3g50280* | transferase family protein similar to anthranilate N-hydroxycinnamoyl/benzoyltransferase, | 2.01 |
|  | *At5g07460* | peptide methionine sulfoxide reductase, putative | 2.00 |
|  | *At4g25850* | oxysterol-binding family protein | -2.00 |
|  | *At4g09260* | hypothetical protein | -2.01 |
|  | *At5g10830* | embryo-abundant protein-related | -2.02 |
|  | *At1g14890* | invertase/pectin methylesterase inhibitor family protein | -2.02 |
|  | *At2g33450* | 50S ribosomal protein L28, chloroplast (CL28) | -2.03 |
|  | *At5g11410* | protein kinase family protein | -2.03 |
|  | *At3g61920* | expressed protein | -2.04 |
|  | *At5g53820* | expressed protein | -2.04 |
|  | *At3g25250* | protein kinase family protein | -2.05 |
|  | *At3g11580* | DNA-binding protein, putative | -2.08 |
|  | *At2g35080* | expressed protein | -2.09 |
|  | *At4g01130* | acetylesterase, putative similar to lanatoside 15'-O-acetylesterase | -2.10 |
|  | *At5g55050* | GDSL-motif lipase/hydrolase family protein | -2.14 |
|  | *At2g47870* | glutaredoxin family protein | -2.14 |
|  | *At5g02540* | short-chain dehydrogenase/reductase (SDR) family protein | -2.14 |
|  | *At5g63790* | no apical meristem (NAM) family protein | -2.16 |
|  | *At4g01575* | serine protease inhibitor, Kazal-type family protein | -2.19 |
|  | *At5g14730* | expressed protein | -2.23 |
|  | *At4g01870* | tolB protein-related | -2.29 |
|  | *At2g30400* | ovate family protein | -2.30 |
|  | *At2g16630* | proline-rich family protein | -2.32 |
|  | *At2g38400* | alanine--glyoxylate aminotransferase, putative | -2.37 |
|  | *At1g15010* | expressed protein | -2.43 |
|  | *At2g22350* | RNase H domain-containing protein | -2.66 |
|  | *At3g14660* | cytochrome P450, putative | -2.69 |
|  | *At2g17280* | phosphoglycerate/bisphosphoglycerate mutase family protein | -2.73 |
|  | *At3g28500* | 60S acidic ribosomal protein P2 (RPP2C) | -2.80 |
|  | *At2g47880* | glutaredoxin family protein | -3.00 |
|  | *At4g08555* | expressed protein | -3.30 |
|  | *At2g24560* | GDSL-motif lipase/hydrolase family protein | -3.34 |
|  | *At2g37870* | protease inhibitor/seed storage/lipid transfer protein (LTP) family protein | -3.35 |
|  | *At1g03070* | expressed protein | -4.05 |
|  | *At1g72660* | developmentally regulated GTP binding protein (DRG1) | -6.60 |
