# Supplementary material for: Dynamics of Membrane Potential Variation and Gene Expression Induced by Spodoptera littoralis, Myzus persicae, and Pseudomonas syringae in Arabidopsis
Source: PLoS One. 2012 Oct 30;7(10):e46673. doi: 10.1371/journal.pone.0046673 (PMC3484130; doi:10.1371/journal.pone.0046673)
Supplement: Table S3 — Arabidopsis thaliana genes commonly expressed at the time of Vm depolarization (5 h) upon Myzus persicae herbivory. (DOCX) [file pone.0046673.s003.docx]

**Supporting Table S3.** *Arabidopsis thaliana* genes commonly expressed at the time of Vm depolarization (5 h) upon *Myzus persicae* herbivory. Values are expressed as fold change with respect to controls (P<0.05). AGI, Arabidopsis Genome Initiative gene index.

| **GO Category** | **AGI** | **Short description** | **FC (P<0.05)** |
| --- | --- | --- | --- |
| flavonoid biosynthetic process | *At5g42800* | dihydroflavonol reductase (DFR) | -30.81 |
|  | *At1g03940* | similar to anthocyanin 5-aromatic acyltransferase from *Gentiana triflora* | -28.04 |
|  | *At4g22870* | leucoanthocyanidin dioxygenase, putative | -24.51 |
|  | *At4g22880* | anthocyanidin synthase (ANS) | -17.06 |
|  | *At3g55120* | chalcone-flavanone isomerase (CFI) | -2.97 |
|  | *At5g05270* | chalcone-flavanone isomerase family protein | -3.33 |
|  | *At1g53520* | chalcone-flavanone isomerase family protein | 5.68 |
|  | *At3g51240* | flavanone 3-hydroxylase (F3H) | -5.81 |
|  | *At5g07990* | CYP75B1, flavonoid 3' hydroxylase. | -16.89 |
|  | *At3g29590* | malonyl-CoA:anthocyanidin 5-O-glucoside-6"-O-malonyltransferase (5MAT) | -13.76 |
|  | *At1g56650* | MYB75, involved in anthocyanin metabolism | -5.81 |
|  | *At5g35550* | MYB123, a key determinant in the proanthocyanidin accumulation | -2.81 |
|  | *At2g45400* | involved in brassinosteroid metabolic pathway (BEN1) | 3.35 |
| fatty acid biosynthetic process | *At1g06360* | Δ-9 desaturase-like 5 protein | 22.36 |
|  | *At2g29980* | fatty acid desaturase (FAD3) | 2.57 |
|  | *At3g15850* | fatty acid desaturase (FAD5) | 2.82 |
|  | *At3g11170* | fatty acid desaturase (FAD7) | 2.04 |
|  | *At5g05580* | fatty acid desaturase (FAD8) | 6.34 |
|  | *At2g15090* | 3-ketoacyl-CoA synthase (KCS8) | 3.01 |
|  | *At4g34250* | 3-ketoacyl-CoA synthase (KCS16) | 3.96 |
|  | *At4g25050* | acyl carrier protein (ACP4) | 3.71 |
|  | *At5g16390* | biotin carboxyl carrier protein (BCCP1) | 2.04 |
|  | *At5g15530* | biotin carboxyl carrier protein (BCCP2) | 2.07 |
|  | *At4g00400* | glycerol-3-phosphate acyltransferase (GPAT8) | 2.04 |
|  | *At1g20510* | 4-coumarate-CoA ligase-like (4CLL5) | -2.43 |
| plant-type cell wall modification and loosening and syncytium formation | *At2g20750* | beta-expansin (EXPB1) | 5.83 |
|  | *At4g17030* | Expansin-related (EXLB1) | -2.66 |
|  | *At1g69530* | Expansin (EXP1) | 5.53 |
|  | *At4g38400* | Expansin (EXPL2) | 2.36 |
|  | *At2g37640* | Expansin (EXP3) | 3.88 |
|  | *At3g29030* | Expansin (EXP5) | 2.00 |
|  | *At2g28950* | Expansin (EXP6) | 2.35 |
|  | *At2g40610* | Expansin (EXP8) | 30.70 |
|  | *At1g26770* | Expansin (EXP10) | 2.80 |
|  | *At1g20190* | Expansin (EXP11) | 4.67 |
|  | *At2g03090* | Expansin (EXP15) | 3.96 |
| oligopeptide transport | *At4g24120* | oligopeptide transporters similar to the yellow stripe locus of maize (YSL1) | -3.76 |
|  | *At4g16370* | oligopeptide transporter OPT family protein | 2.03 |
|  | *At1g09930* | oligopeptide transporter OPT family protein | 2.68 |
|  | *At1g22550* | proton-dependent oligopeptide transport (POT) family protein | -3.28 |
|  | *At1g22570* | proton-dependent oligopeptide transport (POT) family protein | -2.83 |
|  | *At1g72120* | proton-dependent oligopeptide transport (POT) family protein | -2.64 |
|  | *At3g47960* | proton-dependent oligopeptide transport (POT) family protein | -2.31 |
|  | *At5g55930* | proton-dependent oligopeptide transport (POT) family protein | -2.30 |
|  | *At1g33440* | proton-dependent oligopeptide transport (POT) family protein | 2.02 |
|  | *At3g45680* | proton-dependent oligopeptide transport (POT) family protein | 2.18 |
|  | *At1g52190* | proton-dependent oligopeptide transport (POT) family protein | 2.62 |
|  | *At3g45710* | proton-dependent oligopeptide transport (POT) family protein | 2.69 |
|  | *At1g69870* | general substrate transporter | -3.61 |
| multidrug transport | *At1g15520* | ABC transporter (PDR12) | -6.77 |
|  | *At4g21910* | antiporter/ drug transporter, Multi antimicrobial extrusion protein (MATE) | -4.86 |
|  | *At5g52050* | MATE efflux family protein | -4.13 |
|  | *At4g38380* | MATE efflux family protein | -2.69 |
|  | *At1g61890* | MATE efflux family protein | -2.69 |
|  | *At2g04100* | MATE efflux family protein | -2.58 |
|  | *At3g21690* | MATE efflux family protein | -2.02 |
|  | *At5g49130* | MATE efflux family protein | 2.60 |
|  | *At2g04050* | MATE family transporter related protein (ATDTX1) | -2.45 |
|  | *At2g04080* | MATE family transporter related protein (ATDTX1) | -2.21 |
|  | *At5g44050* | MATE family transporter related protein, antiporter | -2.05 |
|  | *At4g22790* | MATE family transporter related protein, ZF14 transporter | -2.04 |
| response to chitin | *At1g19050* | Arabidopsis response regulator (ARR7) | 3.95 |
|  | *At3g18710* | U-box domain-containing protein similar to immediate-early fungal elicitor protein CMPG1 | 2.26 |
|  | *At2g24570* | WRKY17 | 2.21 |
|  | *At4g31800* | WRKY18 | -4.44 |
|  | *At1g80840* | WRKY40 | -5.19 |
|  | *At5g49520* | WRKY48 | -7.42 |
|  | *At4g23810* | WRKY53 | -8.69 |
|  | *At2g25000* | WRKY60 | -2.63 |
|  | *At1g74650* | MYB31 | -10.14 |
|  | *At5g67300* | MYB44 | -2.41 |
|  | *At5g59780* | MYB59 | -3.29 |
|  | *At2g17040* | ANAC036 | -3.24 |
|  | *At3g44350* | ANAC061 | -2.11 |
|  | *At2g40140* | zinc finger family protein (CZF1) | -2.07 |
|  | *At3g55980* | CZF1/ZFAR1 | -2.84 |
|  | *At3g19580* | zinc finger protein (AZF2) | -2.20 |
|  | *At5g43170* | zinc finger protein (AZF3), transcriptional repressor | -4.16 |
|  | *At1g27730* | Cys2/His2-type zinc-finger proteins (STZ), transcriptional repressor | -4.69 |
|  | *At4g17500* | ethylene response factor (ERF1) | -2.63 |
|  | *At2g44840* | ethylene response factor (ERF13) | -4.62 |
|  | *At4g34410* | ethylene redox responsive transcription factor (RRTF1) | -7.99 |
|  | *At4g28140* | DREB subfamily A-6 of ERF/AP2 | -2.38 |
|  | *At3g50260* | DREB subfamily A-5 of ERF/AP2 (DEAR19) | -3.21 |
|  | *At4g17230* | scarecrow-like TF (SCL13) | -2.15 |
|  | *At4g39070* | salt tolerance homolog (STH2) TF | -3.64 |
|  | *At2g35930* | Armadillo-like helical U-box domain-containing protein | -4.50 |
|  | *At5g27420* | RING-H2 finger protein (ATL5H) | -4.87 |
|  | *At4g29110* | Unknown protein | -2.76 |
| response to wounding | *At2g34810* | FAD-binding domain-containing oxidoreductase protein | -3.46 |
|  | *At2g30020* | AP2C1, a MAPK phosphatase that negatively regulates MPK4 | -2.72 |
|  | *At4g16740* | (*E*)-β-ocimene synthase (TPS03) | -2.59 |
|  | *At1g06160* | ethylene response factor (AP2/ERF59) | -2.59 |
|  | *At4g16990* | resistance to *Leptosphaeria maculans* (RLM3) | 2.18 |
|  | *At5g57170* | macrophage migration inhibitory factor family protein / MIF | 2.26 |
|  | *At1g05630* | inositol polyphosphate 5-phosphatase (5PTASE13) | 2.71 |
| response to auxin stimulus | *At1g15580* | auxin induced protein (IAA5) | 3.29 |
|  | *At1g52830* | auxin induced protein (IAA6) | 6.44 |
|  | *At4g14550* | auxin induced protein (IAA14) | 2.57 |
|  | *At3g15540* | auxin induced protein (IAA19) | 3.93 |
|  | *At2g38120* | auxin influx transporter (AUX1) | 2.04 |
|  | *At3g23050* | auxin resistant (AXR2) | 2.36 |
|  | *At4g38850* | auxin-responsive protein , Small Auxin Upregulated (SAUR) (SAUR15) | 4.00 |
|  | *At5g18010* | auxin-responsive protein (SAUR19) | 3.75 |
|  | *At5g18020* | auxin-responsive protein (SAUR20) | 3.88 |
|  | *At5g18050* | auxin-responsive protein (SAUR22) | 3.68 |
|  | *At5g18060* | auxin-responsive protein (SAUR23) | 3.66 |
|  | *At3g03850* | auxin-responsive protein (SAUR26) | 6.38 |
|  | *At3g03820* | auxin-responsive protein (SAUR29) | 7.70 |
|  | *At1g29510* | auxin-responsive protein (SAUR68) | 5.38 |
|  | *At4g34770* | auxin-responsive protein, SAUR-like | 10.35 |
|  | *At4g34790* | auxin-responsive protein, SAUR-like | 6.95 |
|  | *At1g29500* | auxin-responsive protein, SAUR-like | 5.16 |
|  | *At4g38860* | auxin-responsive protein, SAUR-like | 5.05 |
|  | *At1g29450* | auxin-responsive protein, SAUR-like | 4.84 |
|  | *At4g34760* | auxin-responsive protein, SAUR-like | 4.47 |
|  | *At1g29440* | auxin-responsive protein, SAUR-like | 3.75 |
|  | *At4g34810* | auxin-responsive protein, SAUR-like | 3.49 |
|  | *At4g34800* | auxin-responsive protein, SAUR-like | 3.21 |
|  | *At5g18030* | auxin-responsive protein, SAUR-like | 3.03 |
|  | *At1g29460* | auxin-responsive protein, SAUR-like | 2.96 |
|  | *At4g38840* | auxin-responsive protein, SAUR-like | 2.74 |
|  | *At2g06850* | endoxyloglucan transferase (EXGT-A1) | 3.67 |
|  | *At2g47750* | indole-3-acetic acid-amido synthetase (GH3.9) | 2.70 |
|  | *At1g70000* | MYB, response to auxin stimulus | -2.23 |
|  | *At1g74430* | MYB95 | -2.71 |
|  | *At5g63310* | nucleotide diphosphate kinase (NDPK2), modulation of auxin transport | 2.24 |
| response to gibberellin stimulus | *At1g15550* | gibberellin 3 beta-hydroxylase (GA3OX1) | 2.57 |
|  | *At4g25420* | gibberellin 20-oxidase (GA20OX1) | 2.61 |
|  | *At5g15230* | gibberellin-regulated protein (GASA4) | 7.04 |
|  | *At3g02885* | gibberellin-regulated protein (GASA5) | -18.21 |
|  | *At1g74670* | gibberellin-regulated protein (GASA6) | 24.08 |
|  | *At2g14900* | gibberellin-regulated protein | 3.76 |
|  | *At1g22690* | gibberellin-regulated protein | 2.72 |
|  | *At5g59845* | gibberellin-regulated protein | 2.64 |
|  | *At3g05120* | gibberellin receptor (ATGID1A) | -2.22 |
|  | *At5g17490* | DELLA (RGL3) | -2.72 |
|  | *At2g16720* | MYB7 | -4.10 |
|  | *At4g25000* | α-amylase (AMY1) | -5.31 |
| response to jasmonic acid stimulus | *At2g06050* | 12-oxophytodienoate reductase (OPR3) | -3.22 |
|  | *At3g25780* | allene oxide cyclase (AOC3) | -3.07 |
|  | *At3g48520* | CYP94B3, jasmonoyl-isoleucine-12-hydroxylase that catalyzes the formation of 12-OH-JA-Ile from JA-Ile | -13.94 |
|  | *At2g39770* | GDP-mannose pyrophosphorylase, cytokinesis defective (CYT1) | -2.03 |
|  | *At1g19180* | Jasmonate-Zim-Domain Protein (JAZ1) | -2.88 |
|  | *At1g74950* | Jasmonate-Zim-Domain Protein (JAZ2) | -2.81 |
|  | *At3g17860* | Jasmonate-Zim-Domain Protein (JAZ3) | -2.04 |
|  | *At1g17380* | Jasmonate-Zim-Domain Protein (JAZ5) | -5.18 |
|  | *At1g72450* | Jasmonate-Zim-Domain Protein (JAZ6) | -3.44 |
|  | *At2g34600* | Jasmonate-Zim-Domain Protein (JAZ7) | -3.80 |
|  | *At1g70700* | Jasmonate-Zim-Domain Protein (JAZ9) | -4.82 |
|  | *At5g13220* | Jasmonate-Zim-Domain Protein (JAZ10) | -6.72 |
|  | *At3g16470* | Jasmonic Acid Responsive (JR1) | -8.80 |
|  | *At4g23600* | Jasmonic Acid Responsive (JR2) | -3.12 |
|  | *At1g17420* | Lipoxygenase (LOX3) | -3.60 |
|  | *At1g72520* | Lipoxygenase (LOX4) | -4.07 |
|  | *At1g32640* | MYC2 | -2.98 |
|  | *At1g18710* | MYB47 | -3.70 |
|  | *At4g05100* | MYB74 | -3.49 |
|  | *At2g02990* | ribonuclease T2 (RNS1) | -10.38 |
|  | *At3g52400* | syntaxin of plants (SYP122) | -4.55 |
|  | *At1g72260* | thionin (THI2.1) | -18.70 |
