# Supplementary material for: Dynamics of Membrane Potential Variation and Gene Expression Induced by Spodoptera littoralis, Myzus persicae, and Pseudomonas syringae in Arabidopsis
Source: PLoS One. 2012 Oct 30;7(10):e46673. doi: 10.1371/journal.pone.0046673 (PMC3484130; doi:10.1371/journal.pone.0046673)
Supplement: Table S6 — Validation of microarray data. (DOCX) [file pone.0046673.s006.docx]

**Supporting Table S6**. Validation of microarray data. The length of PCR products ranged from 92 to 195 bp.

| Organism | Regulation | AGI code | Short description | Microarray FC | qPCR FC |
| --- | --- | --- | --- | --- | --- |
| *Spodoptera*  *littoralis* | Up regulated | At1g14880 | Plant cadmium resistance 1 (PCR1) | 7,39 | 10,31±2,84 |
|  | Down regulated | At1g52560 | HSP20-like chaperones superfamily | -36,21 | -34,21±0,004 |
| *Pseudomonas*  *syringae* | Up regulated | At4g21840 | Methionine reductase B8 (MSRB8) | 36,60 | 15,69±2,34 |
|  | Down regulated | At2g15490 | UDP-glycosyltransferase 73B4 (UGT73B4) | -16,45 | -21,42±0,013 |
| *Myzus*  *persicae* | Up regulated | At5g20630 | Germin-like protein (GER3) | 46,29 | 17,41±1,09 |
|  | Down regulated | At5g10625 | Flowering promoting factor 1 | -29,44 | -29,23±0,002 |
